# Confronting stresses affecting olive cultivation from the holobiont perspective

**DOI:** 10.3389/fpls.2023.1261754

**Published:** 2023-11-06

**Authors:** Martina Cardoni, Jesús Mercado-Blanco

**Affiliations:** Departamento de Microbiología del Suelo y la Planta, Estación Experimental del Zaidín, Consejo Superior de Investigaciones Científicas (CSIC), Granada, Spain

**Keywords:** biocontrol, co-occurrence network analysis, *Olea europaea*, olive microbiome, plant functional traits, root architecture, verticillium wilt of olive, *Xylella fastidiosa*

## Abstract

The holobiont concept has revolutionized our understanding of plant-associated microbiomes and their significance for the development, fitness, growth and resilience of their host plants. The olive tree holds an iconic status within the Mediterranean Basin. Innovative changes introduced in olive cropping systems, driven by the increasing demand of its derived products, are not only modifying the traditional landscape of this relevant commodity but may also imply that either traditional or emerging stresses can affect it in ways yet to be thoroughly investigated. Incomplete information is currently available about the impact of abiotic and biotic pressures on the olive holobiont, what includes the specific features of its associated microbiome in relation to the host’s structural, chemical, genetic and physiological traits. This comprehensive review consolidates the existing knowledge about stress factors affecting olive cultivation and compiles the information available of the microbiota associated with different olive tissues and organs. We aim to offer, based on the existing evidence, an insightful perspective of diverse stressing factors that may disturb the structure, composition and network interactions of the olive-associated microbial communities, underscoring the importance to adopt a more holistic methodology. The identification of knowledge gaps emphasizes the need for multilevel research approaches and to consider the holobiont conceptual framework in future investigations. By doing so, more powerful tools to promote olive’s health, productivity and resilience can be envisaged. These tools may assist in the designing of more sustainable agronomic practices and novel breeding strategies to effectively face evolving environmental challenges and the growing demand of high quality food products.

## 1 Introduction: importance and cultivation range of the olive holobiont

Olive is a member of the *Oleaceae*, a botanical family including around 30 genera and 600 species (Fogher et al., 2010). The *Olea* L. genus consists of 33 species of evergreen shrubs and trees with ample natural distribution in the warm-temperate areas of the world, *Olea europaea* L. being the only cultivated species. Within the subspecies *europaea*, two distinct varieties coexist, namely the wild olive or oleaster (*O. europaea* subsp. *europaea* var. *sylvestris*) and the cultivated olive (*O. europaea* subsp. *europaea* var. *europaea*). Extensive multilocal selection practices, coupled with deliberate backcrossing between wild and cultivated olive plants, have contributed significantly to the vast assortment of cultivars that are presently available (Besnard et al., 2001).

**Figure 1:**
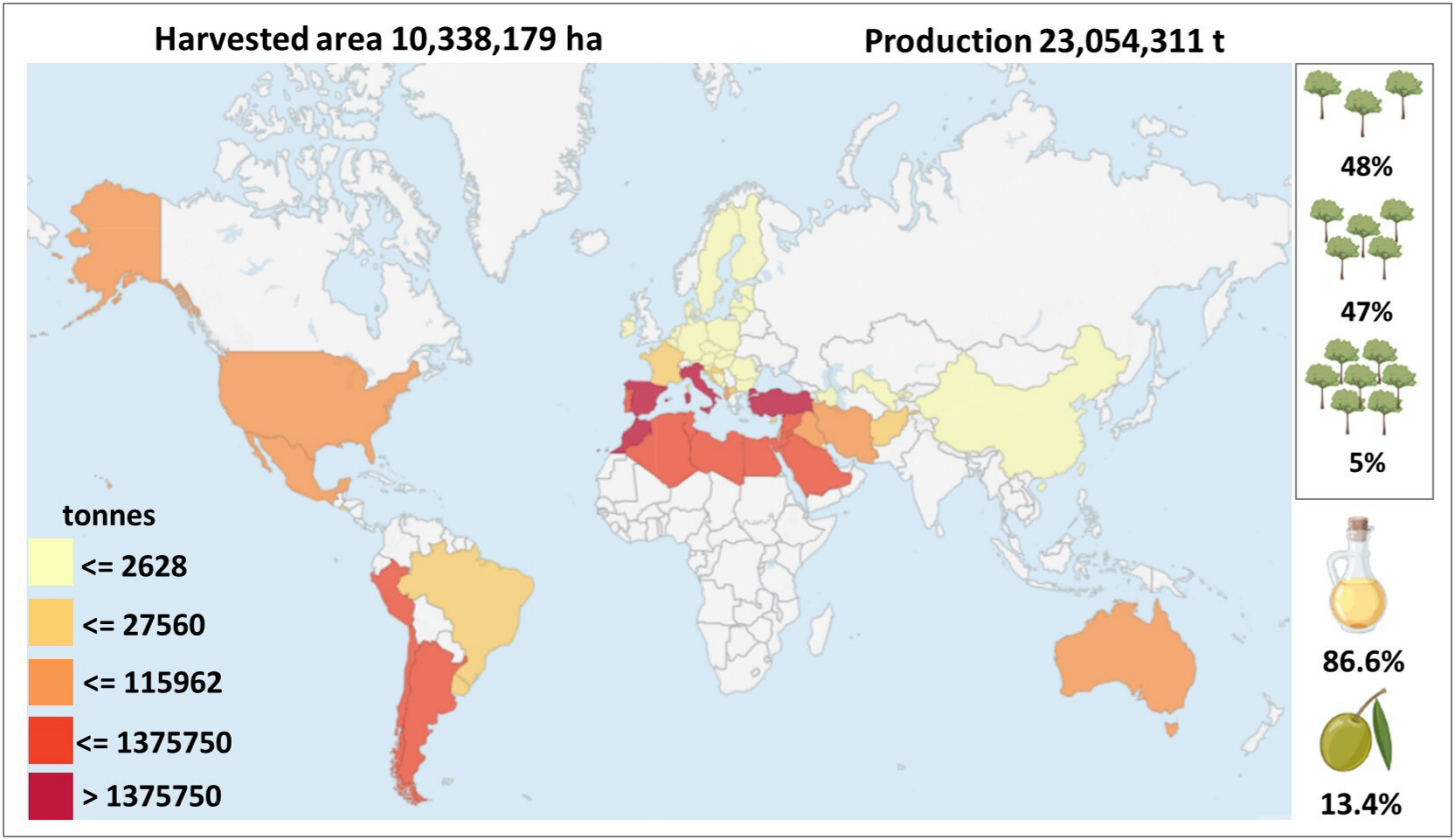
Acreage and productions of olive oil and table olives at world scale in 2020. The image was created with data from FAOSTAT 2021 (production quantities of olives by country). The tree drawings on the right show, in descending order, the percentages of the different olive cultivation systems: traditional (less than 140 tress/ha), medium-density (from 140 to 399 trees/ha) and high-density (over 400 trees/ha) (Russo et al., 2016). The drawings of the oil bottle and the olive below represent the percentages of olive production dedicated to oil and table olives, respectively.

Olive is not only relevant as source of important nutritional products, but also for shaping and safeguarding the landscape while influencing the human rural lifestyle (Vilar et al., 2018). At present time, olive is cultivated in nearly 60 countries along 5 continents. However, its consumption extends to a total of 179 countries (Vilar et al., 2018). This scenario highlights that the olive sector is based on an extremely localized production and a globally dispersed demand. Global olive cultivation spans approximately 10.4 million hectares (ha) (**Figure 1**). The Mediterranean area accounts for around 9 million ha, hosting nearly 98% of olive oil and 80% of table olive production. (IOC, https://www.internationaloliveoil.org/; FAOSTATS, https://www.fao.org/faostat/en/#data/QCL) (**Figure 1**). It is worth noting that over the past decades, olive cultivation has seen a steady expansion into (semi)arid regions, including countries such as Turkey, Syria, and Saudi Arabia (Tubeileh et al., 2009). This scenario has the potential to significantly influence the future of olive production worldwide. Indeed, recent underwhelming olive harvests in traditional major European producers may represent an opportunity for olive oil manufacturers in these areas, thereby reshaping the dynamics of the olive oil industry.

To satisfy the rising global demand of olive products, each growing region has to respond by including new olive orchards in the existing agroecological zone and/or expanding to new agroecological regions, or by changing methods of cultivation and orchard managements. Within this scenario, 162,000 ha of olive groves are planted every year to fulfill the needs of the market (Vilar et al., 2018). Furthermore, olive cultivation is experiencing a revolution due to changes from a traditional cropping model to high-density plantation systems linked to the above-mentioned increasing demand (Sanzani et al., 2012) (**Figure 1**).

**Figure 2:**
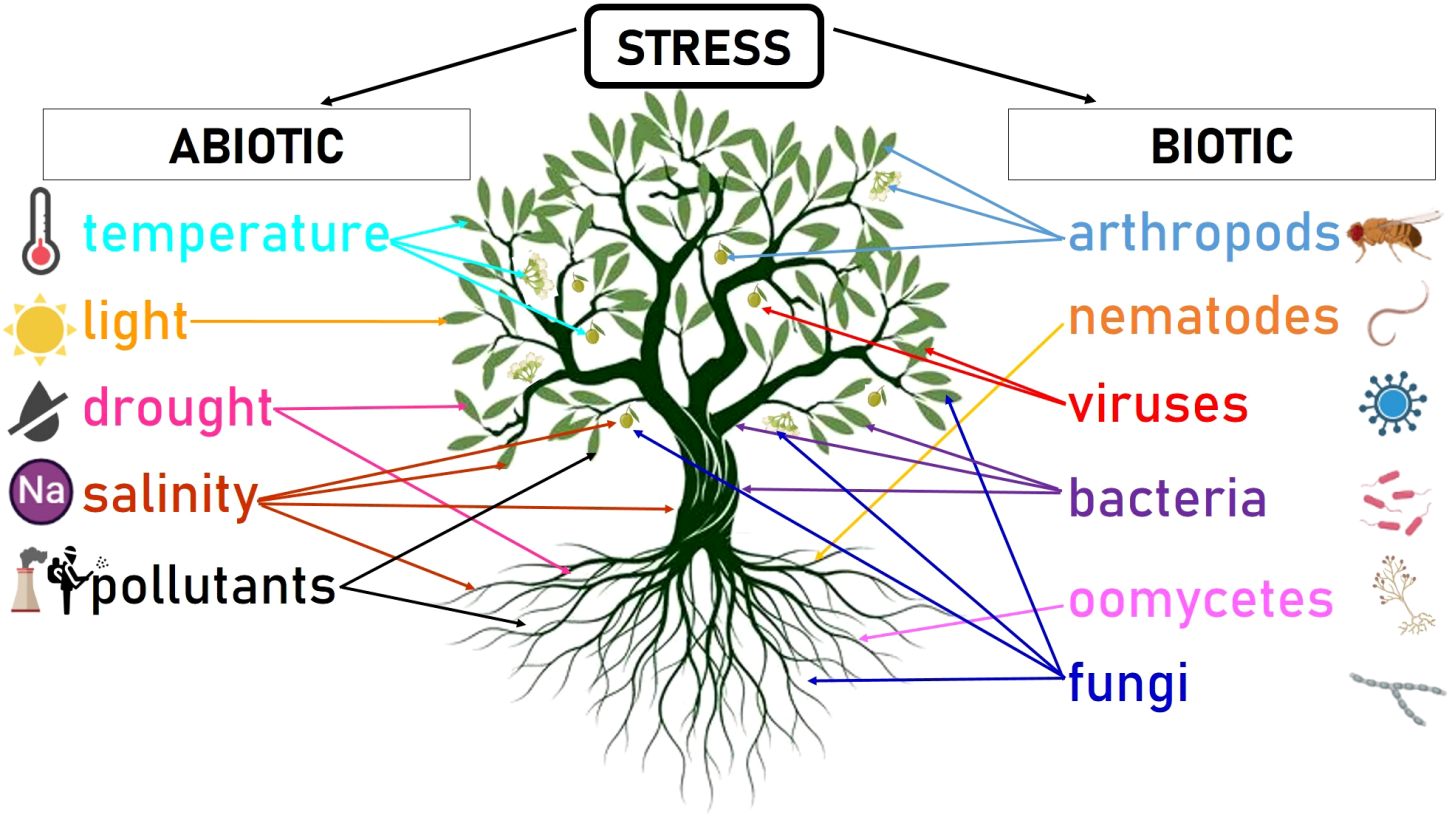
Schematic summary of the most relevant abiotic and biotic stresses that can affect olive trees. Arrows point to the plant parts that can be affected by the particular stress (i.e. leaf, flower, fruit, trunk, stem, root). The figure was created with BioRender.com.

This new concept of olive orchard can potentially impact the exposure of trees to a range of traditional and emerging stresses. Stress, defined as any detrimental effect experienced by an organism, can be categorized as internal or external. Internal stresses arise from mutations or aberrant cell divisions that can produce metabolic alterations. On the other hand, external stresses may originate from biotic sources (such as pests and pathogens) or abiotic factors (such as climate conditions or soil characteristics) (Macedo, 2012) (**Figure 2**).

Despite its relevance and widespread cultivation, no studies are available about the effect that abiotic and/or biotic stresses have on olive from a holistic perspective; that is, considering this tree as a metaorganism composed of the host and its associated microbiome, the so-called holobiont. Therefore, a comprehensive understanding of the olive holobiont, including anatomy, morphology, physiology, genetics, biochemistry and its closely-related microbial communities is crucial to fully exploit field performance, health, fitness, resilience and productivity of this woody crop. The aims of the present review article are to provide an up-to-date appraisal on: 1) the abiotic and biotic stresses affecting olive cultivation, 2) the olive-associated microbiome, and 3) the biotic and biotic factors that may compromise microbiome structure, composition and interactions. Finally, we discuss the importance to analyse the olive plant under the holobiont perspective.

## 2 An overview of the most important abiotic stresses affecting the olive tree

Olive trees are optimally grown in regions located between latitudes 30° and 45° of both hemispheres in Mediterranean-type climate regions (Montes-Osuna and Mercado-Blanco, 2020). Ideal weather conditions for olive cultivation are warm and sunny summers, and cold and rainy winters. Temperatures during the cold season should not go below -10°C during a prolongued period of time. The rain must be concentrated in autumn-winter since olive is primarily an anemophilous species. Thus, areas with high humidity levels during the flowering period (late spring) are not suitable (Mancuso, 2000). Abiotic disorders, particularly in the case of long-term cultivated plants like olive trees, tend to be poorly understood and frequently overlooked, often due to the minimal care they receive over extended periods (Sanzani et al., 2012). However, farmers applying either traditional or novel cultivation systems are increasingly conscious of the relevance of abiotic factors due to their adverse impact on yield and quality. Additionally, the capacity of certain abiotic stresses to affect resistance levels against parasitic and non-parasitic diseases is well recognized (Graniti et al., 2011; Sanzani et al., 2012) (**Figure 2**). In this review, we focused on the most common abiotic stresses affecting olive trees in the Mediterranean Basin: temperature, drought, salinity, and pollutants.

### 2.1 Temperature

Climatic constraints influence olive’s global distribution. Olive cultivation is absent beyond 45° north and south latitudes due to its vulnerability to temperatures around -12°C (Mancuso, 2000). Low temperatures can influence the physiology of the plant, leading to effects like chilling injury, or by causing frost damage that varies in severity depending on the timing of the occurrence within the year. Leyva-Pérez and co-authors studied the gene expression pattern of ‘Picual’ plants exposed to cold stress, identifying more than 6,300 unigenes differentially expressed in response to cold, which were subsequently categorized in three types of responses (i.e. short term, early long term and late long term) leading to cold acclimation (Leyva-Pérez et al., 2015). In both pathological and ecological contexts, high temperatures have been found to be less limiting compared to low temperatures. Instances of high temperature damage are rare and often coinciding with drought, excessive light, strong winds, and low humidity. Certain olive varieties acclimated to elevated temperatures (e.g. 40°C) maintain 70-80% photosynthetic activity (Bongi et al., 1987), enabling cultivation in desert areas. However, damages due to high temperatures are estimated to increase due to greenhouse gas accumulation, driving global temperature rise (Gibelin and Déqué, 2003). At high temperatures, leaves turn yellow and roll up, their tips and margins may turn brown, and eventually defoliation takes place (Sanzani et al., 2012). Furthermore, persistent heat (above 33°C) during flowering diminishes fruit set (Graniti et al., 2011). Drupes also suffer in summer, displaying redness or dried spots (Sanzani et al., 2012). Sunburn, a form of abiotic stress resulting from intense heat or direct exposure to sunlight on olive tree limbs, is another challenge associated with high temperatures. Sunburn can negatively impact growth, causing a gradual decline in tree health and even the death of young olive plants (Sergeeva and Spooner-Hart, 2011).

### 2.2 Drought

Similarly to numerous Mediterranean woody species adapted to semi-arid conditions, olive trees exhibit tolerance to soil water scarcity. In fact, olive has morphological and physiological characteristics typical of a xerophyte, although it can be cultivated in regions with abundant water supply. Olive plants show several morphological and biochemical adaptations to stand dehydration and water shortage (e.g. low leaf conductance, deep roots, high root length density, osmotic adjustment of cell contents, changes in cell wall elasticity) (Sanzani et al., 2012). However, olive groves may suffer under persistent periods of water deficiency. Drought stress has detrimental effects on the growth parameters of olive trees, predominantly in young plantations. Water deficit greatly affects shoot growth, subsequently impacting the non-bearing fruit period of the trees which can result in important economic loss. Furthermore, drought adversely affects the water content and succulence of the leaves, as well as the carbon assimilation rate, stomatal conductance, and water use efficiency (Denaxa et al., 2012). Water scarcity additionally diminishes the quality and organoleptic characteristics of virgin olive oil, particularly its phenol content (Sanzani et al., 2012). A recent study highlighted the capacity of olive trees to enlist specific bacteria, for which the term ‘aridity winners’ was coined, capable of enduring drought and arid conditions. It has been suggested that these microorganisms could play a significant role in helping plants adapt to sudden changes caused by climate change (Marasco et al., 2021).

### 2.3 Salinity

Salinity, whether natural or induced by irrigation, poses challenges in semi-arid olive-growing regions. Sodium chloride (NaCl), the most common soluble salt, negatively impacts shoot growth, fruit yield, and alters photosynthesis, inducing leaf morphological changes (Bazakos et al., 2015). The olive tree is considered moderately tolerant to salinity although significant differences in salt tolerance have been registered among cultivars (Chartzoulakis et al., 2002; Kchaou et al., 2010). Young trees are less salt-tolerant than mature ones. Employing tolerant cultivars, along with strategic irrigation, improved soil drainage, and light tillage to curb evaporation, is advised in salinity-affected sites (Graniti et al., 2011). Utilizing plant growth-promoting microorganisms (PGPM) has been suggested as a strategy to mitigate or reduce the impact of salinity stress. Indeed, some PGPMs have the ability to stimulate mechanisms related with salt stress tolerance in plants [i.e. production of extracellular polymeric substances and antioxidants, nitrogen fixation, phytohormone biosynthesis, or the activity of 1-aminocyclopropane-1-carboxylic acid (ACC) deaminase (ACD)] (Kumar et al., 2020). However, better tolerance to salinity was not found in young olive plants inoculated with two different PGPMs (*Pseudomonas* sp. PICF6 and *Pseudomonas simiae* PICF7), regardless of whether (PICF6) or not (PICF7) they displayed ACD activity (Montes-Osuna et al., 2021b).

### 2.4 Pollutants

Over the past decades a consistent increase of pollutants in water, soil and air caused mainly by industrial and agricultural activities has taken place. The alteration of the atmospheric composition by gases emitted by human activity is probably the most harmful, since it can seriously affect plant health and productivity. Among key air pollutants, carbon dioxide (CO_2_), ozone (O_3_), sulphur dioxide (SO_2_) and fluorides (F^-^) are notable (Sebastiani et al., 2002). While stricter fuel regulations have reduced SO_2_ prevalence, it remains impactful when combined with other pollutants, such as oxides of nitrogen, fluorides, and ozone. Indeed, its impact is more severe when in combination with these compounds that amplify its ability to promote the plant stomata and accelerate the water loss (Varshney et al., 2009).

The use of treated wastewaters for irrigation is another source of concern. This practice has gained traction in recent years, driven by the scarcity of water resources particularly in regions characterized by low rainfall. In these regions, farmers have adopted strategies incorporating marginal water sources, like treated wastewater, for supplementary irrigation (Al-Habahbeh et al., 2021). These waters pose a challenge due to potential soil pollution and phytotoxicity from salt and heavy metals accumulation (e.g. Pb, Cd, Mn, Cu). Olive trees are considered important heavy metal bioaccumulators in different plant parts for Cu, Pb and Zn (Wilson and Pyatt, 2007). Thus, reducing heavy metal accumulation in water and soil is increasingly crucial to prevent plant uptake and subsequent entry into the food chain.

Contamination by pesticides is also problematic in the Mediterranean area. Certainly, olive orchards can be affected by this type of pollution (Calderón et al., 2016). Furthermore, these chemicals can be accumulated in the soil, the extent of contamination being influenced by factors such as the timing (and number) of application, specific characteristics of the pesticide, tree density, and the surface area of the soil covered by vegetation (Calderón et al., 2016). Studies focusing on water monitoring have documented the widespread occurrence of triazine herbicides within olive groves. For this reason, oxyfluorfen and glyphosate, less soluble in water, were introduced as an alternative in olive production. However, some studies have reported dangerous side effects of these chemicals for both the environment and human health (Myers et al., 2016). Moreover, limited information regarding their impacts on olive trees is available. While several studies alert about the effect of pesticides on the biodiversity (i.e. nematodes, spiders, insects, birds) preent in olive orchards (Carpio et al., 2019; Morgado et al., 2020), very little is known about their accumulation in olive organs and the ability of the tree to metabolize and transform these chemicals into less toxic substances. Furthermore, presence (contamination) of herbicides in water can depend on factors such as field application rates and water solubility (Calderón et al., 2015). Nonetheless, there is a scarcity of *in situ* investigations concerning the influence of soil processes and rainfall on soil-applied herbicides, with no research conducted specifically in olive orchards.

## 3 An overview of the most important biotic stresses affecting olive trees

Olive diseases and pests have been historically reported in the Mediterranean area (Graniti et al., 2011). Moreover, the spread of olive cultivation to new regions worldwide and the introduction of cropping innovations aimed to increase productivity and profits can potentially increase the exposure of olive trees to new diseases and pests (**Figure 2**). Besides, there is strong evidence that the increasing concentration of greenhouse gases in the atmosphere occurring on a global scale might have significant effects on plant pathogens (Graniti et al., 2011; Sanzani et al., 2012).

### 3.1 Arthropods

*Bactrocera* (Dacus) *oleae* (Rossi) (*Diptera, Tephritidae*), the olive fruit fly, is a strictly monophagous insect pest feeding exclusively on unripe green fruits (Abdelfattah et al., 2018) (**Table 1**). It can be observed in all countries of the Mediterranean Basin where olive trees are grown. Visible through fruit punctures and larval feeding, the physical harm detrimentally impacts crop yield as well as the quality of both fruit and oil. The female punctures the fruit with the ovipositor thus leaving the egg under the fruit skin. The larva feeds on the mesocarp, causing the drupe to fall from the tree (Barranco Navero et al., 2017). In order to overcome the natural defence of olive plants, in particular the production of secondary metabolites, the olive fruit fly evolved to harbor a vertically transmitted and obligate bacterial symbiont, namely *Candidatus* Erwinia dacicola (Nobre, 2021). It has been also reported that *B. oleae* can facilitate secondary microbial infections caused by different microorganisms (Abdelfattah et al., 2018). Over the past five decades, olive fruit fly management relied on chemical insecticides (e.g. organophosphates, pyrethroids, and spinosad derived from *Saccharopolyspora spinosa*). Yet, extensive insecticide use led to resistance. Alternatives encompass mass trapping, natural enemies, and sterile insect techniques (Corrado et al., 2016).

**Table 1:** Most common arthropods and nematodes affecting olive cultivation worldwide.

Common name	Scientific name	Host plants	Affected plant organs/tissues	Control methods	Selected references
Arthropods					
White louse	*Aspidiotus nerii*	Olive, Carob, Citrus, Ornamental plants	Stem, leaf, fruit	Chemical insecticides	Alexandrakis et al., 1977; Alexandrakis and Benassy, 1981
Olive fruit fly	*Bactrocera oleae*	Olive	Fruit	Chemical insecticides Mass trapping Insect sterilization Biological control	Corrado et al., 2016; Abdelfattah et al., 2018; Nobre, 2021
Olive psyllid	*Euphyllura olivina*	Olive	Flower blossoms and soft growing tissue	Chemical insecticides	Hrncic, 2002; Malumphy, 2011; Barranco Navero et al., 2017
Olive moth	*Prays oleae*	Olive	Leaf, flower, fruit	Biological control	Barranco Navero et al., 2017; Nobre, 2021
Olive bark midge	*Resseliella oleisuga*	Olive, Ash tree, Phyllirea	Stem, trunk	Chemical insecticides Protecting cutting surface of branches	Alvarado et al., 2007; Bejarano-Alcázar et al., 2011
Black scale	*Saissetia oleae*	Olive, Citrus, Ornamental plants	Leaf	Biological control pruning	Abd-Rabou, 2004; Barranco Navero et al., 2017
Leopard moth	*Zeuzera pyrina*	Olive, Pear, Apple	Stem, trunk	Chemical insecticides pruning	Durán et al., 2004; Sabbour, 2017
	*Aceria oleae*	Olive	Leaf	Chemical control Biological control	Leiva et al., 2013
	*Ditrymacus athiasellus*	Olive	Leaf	Chemical control	Gilbert and Mifsud, 2007
Olive bud mite	*Opisthotropis maxwelli*	Olive	Leaf, bud, stem	Chemical control	Gilbert and Mifsud, 2007
	*Oxycenus maxwelli*	Olive	Leaf	Chemical control Biological control	Leiva et al., 2013
Olive rust mite	*Tegolophus hassani*	Olive	Leaf, shoot	Chemical control	Gilbert and Mifsud, 2007
Nematodes					
Spiral nematode	*Helicotylenchus digonicus*	Vegetables, Fruit trees, Cereals, Ornamentals	Root epidermis/root hair	Host plant resistance Crop rotation Biological control	Siddiqui et al., 1973; Talavera and Navas, 2002; Andrássy, 2007
Spiral nematode	*Helicotylenchus neopaxilli*	Olive	Root	Soil/plant material sterilization Chemical control	Inserra et al., 1979; Ali et al., 2014
Spiral nematode	*Helicotylenchus oleae*	Olive	Root	Soil/plant material sterilization Chemical control	Inserra et al., 1979; Palomares Rius et al., 2018
	*Meloidogyne baetica*	Olive	Root	Soil/plant material sterilization	Castillo et al., 2003a; Vovlas et al., 2004; Ali et al., 2014
Root-knot nematode	*Meloidogyne incognita*	Vegetables, Fruit trees, Ornamental	Root epidermis/root hair/root	Soil/plant material sterilization Chemical control Biocontrol (nematophagous fungi) Solarization	Escobar et al., 2015; Ahmed and Omara, 2018; Tapia-Vázquez et al., 2022
Sugarcane eelworm, Javanese root-knot nematode	*Meloidogyne javanica*	Tea, Grapevine, Vegetables, Fruit trees, Cereals, Ornamentals	Root epidermis/root hair/root	Biocontrol (PGPB, nematophagous fungi, predatory nematodes)	Ali et al., 2014; Escobar et al., 2015; Tapia-Vázquez et al., 2022
	*Meloidogyne lusitanica*	Olive	Root	Soil/plant material sterilization	Abrantes and Santos, 1991; Ali et al., 2014
Walnut meadow nematode, Walnut root-lesion nematode	*Pratylenchus vulnus*	Grapevine, Vegetables, Fruit trees, Cereals	Cortex, root	Host plant resistance Chemical control	Zunke, 1990; Ali et al., 2014
	*Rotylenchulus macrosoma*	Olive	Root	Soil/plant material sterilization	Castillo et al., 2003b; Palomares-Rius et al., 2021a; Palomares-Rius et al., 2021b

Another relevant pest of olive trees is the olive moth, *Prays oleae* (Bernard) (*Lepidoptera, Praydidae*) (Nobre, 2021) (**Table 1**). It has three generations a year and larvae feed on diverse olive tree organs: leaves (phillophagous generation), flowers (antophagous generation) and fruits (carpophagous generation) (Nobre, 2021). Weather conditions greatly affect the olive moth. Eggs and larvae exhibit high susceptibility to low humidity and high temperatures, what explain the limitd presence of this pest in hot and arid continental regions (Barranco Navero et al., 2017). The best method to control this insect is the use of natural enemies. For example, effective control of *P. oleae* pupae by spiders living in the canopy of olive groves has been reported (Villa et al., 2020).

*Saissetia oleae* (Olivier) (*Homoptera, Coccidae*), causing olive black scale, is another serious pest able to infest approximately 115 different host plants (Abd-Rabou, 2004). The severity of damages caused by *S. oleae* depends on the level of infestation (**Table 1**), ranging from injuries due to sap extraction to the appearance of honey dew on the leaves, resulting in the spread of sooty mold that causes the reduction of photosynthetic and respiration rates. Afterwards, more severe symptoms emerge, leading from leaf drop to eventual near-complete branch defoliation. Pruning is a good way to fight against this pest (Barranco Navero et al., 2017). Another effective method is biological control through parasites, such as *Hymenoptera*, or predators, such as *Coleoptera, Lepidoptera*, and *Hymenoptera* (Haniotakis, 2005). Other insects that can cause problems depending on specific environmental and/or agronomical conditions are *Euphyllura olivina, Zeuzera pyrina, Aspidiotus nerii*, and *Resseliella oleisuga* (Barranco Navero et al., 2017) (**Table 1**).

Besides insects, mites can also cause damage to olive trees. They inhabit leaves, buds, flowers, and fruits, inducing greenish-yellow markings on mature leaves, distortions on young leaves, dark green indentations, rust patches on buds, and fruit deformities. Thirty species (belonging to the families *Eriophyidae, Tenuipalpidae* and *Tetranychidae)* have been detected in olive trees worldwide (Tzanakakis, 2003). The species *Aceria oleae* and *Oxycenus maxwelli* are present in most of the Mediterranean countries and South Africa (Tzanakakis, 2006). Other three species of eryophid mites have been associated with important economic losses in the Maltese islands: *Opisthotropis maxwelli, Ditrymacus athiasellus* and *Tegolophus hassani* (Gilbert and Mifsud, 2007) (**Table 1**).

### 3.2 Nematodes

Plant-parasitic nematodes (PPN) are soil-borne microscopic animals mainly feeding on root cells by means of a spear-like structure named stylet. The nematodes insert this structure into the plant cells thereby injecting digestion secretions and sucking cell contents. Many species (e.g. *Helicotylenchus digonicus, Meloidogyne javanica, M. incognita* and *Pratylenchus vulnus*) can parasite a broad range of cultivated and wild plants, while others such as *H. oleae, H. neopaxilli, M. baetica, M. lusitanica, Rotylenchulus macrosoma* are specific to olive trees (Castillo et al., 2003b; Ali et al., 2014; Palomares Rius et al., 2018; Palomares-Rius et al., 2021a; Palomares-Rius et al., 2021b) (**Table 1**). Root-knot nematodes (RKN, *Meloidogyne* spp.) are important olive tree pests, particularly in nurseries under favorable irrigation conditions favoring their propagation (Aït Hamza et al., 2017). Most RKN infestations in olive orchards originate from contaminated plant material produced in uncertified nurseries or from the use of nonsterile substrates to cultivate the trees. Nematode infection indirectly harms roots, providing an entry for soil-borne pathogens (e.g. bacteria, fungi) (Ali et al., 2014; Tapia-Vázquez et al., 2022). Notably, nematodes like *M. incognita* and *P. vulnus* often associate with the fungal pathogen *Verticillium dahliae* (Kleb.). Indeed, the presence of nematodes can enhance the symptoms caused by the fungus (Saeedizadeh et al., 2003). Furthermore, nematodes can strongly influence the microbiota structure, feeding on fungi and bacteria (Mercado-Blanco et al., 2018). A useful control method of nematodes is the introduction into substrates of microbial antagonists or PGPM that stimulate plant resistance. Microbial antagonists of PPN species comprise nematophagous fungi that may act by antibiosis, parasitism or predation (Ali et al., 2014). The biological control potential of plant growth promoting bacteria (PGPB) against phytoparasitic nematodes has been studied in species of *Agrobacterium, Arthrobacter, Azotobacter, Clostridium, Desulfovibrio, Pasteuria, Serratia, Burkholderia, Azospirillum, Bacillus, Chromobacterium, Pseudomonas*, and *Corynebacterium, Bacillus* and *Pseudomonas* being the most promising genera (Tapia-Vázquez et al., 2022).

### 3.3 Viruses

At present, fifteen viruses from nine genera across eight families have been identified to be able to affect olive trees. Some viruses have wide effects on various crops, while others are specific to olives: Olive latent ring spot virus (OLRV), the Olive leaf yellowing-associated virus (OLYaV), the Olive latent virus 3 (OLV-3), and the Olive mild mosaic virus (OMMV), a recombinant between OLV-1 and Tobacco necrosis virus D (TNV-D) (Cardoso et al., 2005; Alabdullah et al., 2009; Corrado et al., 2016; Varanda et al., 2018) (**Table 2**). Epidemiology of olive viruses remains largely unknown. The mechanisms most likely favoring their spread are fungus-mediated transmission through the soil and contamination in nurseries (Corrado et al., 2016). Recently, Caruso and co-workers developed a new real-time reverse transcription-loop-mediated isothermal amplification (RT-LAMP) method for OLYaV detection. It exhibits high sensitivity on positive samples, especially on asympomatic olive plants (Caruso et al., 2023). Among the viruses affecting various crops and also identified in olives are the Strawberry latent ring spot virus (SLRSV; family *Secoviridae*, genus *Sadwavius*), the Cherry leaf roll virus (CLRV; family *Secoviridae*, genus *Nepovirus*), Arabis (ArMV), Cucumber (CMV) and Tobacco (TMV) mosaic viruses, categorized as *Nepovirus, Cucumovirus* and *Tobamovirus*, respectively (Caglayan et al., 2011; Martelli, 2013; Corrado et al., 2016) (**Table 2**). Measures for halting virus dissemination and generating virus-free plant propagation material are mostly based on sanitary selection and sanitation (Caglayan et al., 2011). Regrettably, there are few studies on virus eradication from vegetatively propagated olive material. In Italy, promising outcomes were attained for eliminating CLRV and OLYaV through heat therapy and shoot tip culture from infected olive trees (Bottalico, 2002).

**Table 2:** Diseases threatening olive cultivation worldwide.

Disease common name	Disease/organism scientific name	Symptoms	Host plants	Control methods*	Selected references
Viruses					
ArMV	Arabis mosaic virus	Leaf yellowing, deformation of the shoots, bumpy fruit	Birch, elm, ash, elderberry, beech, olive	Sanitary selection Plant material sterilization	Fadel et al., 2005; Caglayan et al., 2011; Corrado et al., 2016
CLRV	Cherry leaf roll virus	Leaf yellowing, fasciation and deformation of the shoots	Woody plants fruit trees, shrubs, olive,	Sanitary selection Hot therapy	Mahy, 2008; Caglayan et al., 2011; Corrado et al., 2016
CMV	Cucumber mosaic virus	Leaf chlorosis, bumpy fruit	Woody plants, fruit trees, vegetables, ornamental plants, citrus tulip, olive	Sanitary selection, Plant material sterilization	Fadel et al., 2005; Caglayan et al., 2011; Corrado et al., 2016
OLRV	Olive latent ringspot virus	Chlorotic/necrotic local lesions, distortion and mottling of upper non-inoculated leaves	Olive, quinoa, globe amaranth	Sanitary selection, Plant material sterilization	Savino and Gallitelli, 1983; Caglayan et al., 2011; Corrado et al., 2016
OLV-1	Olive latent virus 1	Leaf yellowing, fasciation and deformation of the shoots	Benth, olive, citrus, tulip	Sanitary selection, Plant material sterilization	Griego et al., 1996b; Caglayan et al., 2011; Corrado et al., 2016
OLV-2	Olive latent virus 2	Leaf yellowing, fasciation and deformation of the shoots	Olive, castorbean	Sanitary selection, Plant material sterilization	Griego et al., 1996a; Caglayan et al., 2011; Corrado et al., 2016
OLV-3	Olive latent virus 3	Leaf yellowing, fasciation and deformation of the shoots	Benth, olive	Sanitary selection, Plant material sterilization	Alabdullah et al., 2009; Alabdullah et al., 2010; Martelli, 2013
OLYaV	Olive leaf yellowing-associated virus	Leaf yellowing	Olive	Sanitary selection, Hot therapy	Caglayan et al., 2011; Corrado et al., 2016; Fontana et al., 2019
OMMV	Olive mild mosaic virus	Leaf chlorosis and necrosis	Olive, tulip, spinach	Sanitary selection, Plant material sterilization	Gratsia et al., 2012; Varanda et al., 2018
OSLV	Olive semi latent virus	Leaf yellowing, fasciation and deformation of the shoots	Olive	Sanitary selection, Plant material sterilization	Caglayan et al., 2011; Martelli, 2013; Corrado et al., 2016
OVYaV	Olive vein yellowing-associated virus	Leaf yellowing, fasciation and deformation of the shoots	Olive	Sanitary selection, Plant material sterilization	Caglayan et al., 2011; Martelli, 2013
OYMDaV	Olive yellow mottling and decline associated virus	Leaf yellowing, fasciation and deformation of the shoots	Olive	Sanitary selection, Plant material sterilization	Caglayan et al., 2011; Martelli, 2013; Corrado et al., 2016
SLRSV	Strawberry latent ringspot virus	Bumpy fruit	Fruit trees, small fruits, vegetables, weed, ornamental plants, olive	Plant material sterilization	Marte et al., 1986; Faggiolini et al., 2008; Caglayan et al., 2011; Corrado et al., 2016
TMV	Tobacco mosaic virus	Leaf yellowing, fasciation and deformation of the shoots	Field crop, vegetables, ornamental plants, olive	Sanitary selection, Plant material sterilization	Mahy, 2008; Caglayan et al., 2011; Corrado et al., 2016
Bacteria/Phytoplasmas					
Olive knot disease	*Pseudomonas savastanoi* pv. *savastanoi*	Stem and branch hyperplasia	Olive, ash	Chemical control, Avoid plant injury, Pruning tools sterilization, Prune branches with tumors	Ramos et al., 2012; Buonaurio et al., 2015; Moretti et al., 2016; Turco et al., 2022
Olive quick decline syndrome	*Xylella fastidiosa* subsp. *pauca*	Leaf chlorosis, twigs desiccation, decline and die-back	Grapevine, almond, oleander, field crop, citrus, olive	Vectors control/trap	Saponari et al., 2014; Corrado et al., 2016; Saponari et al., 2019
16S-IB	Aster yellows group	Leaf chlorosis, witches broom, flower abortion, decline and die-back	Olive	Antibiotics, Resistant cultivars, Vectors control/trap	Pasquini et al., 2000
16Sr-VIIB	Ash yellow group	Leaf chlorosis, witches broom, flower abortion, decline and die-back	Olive	Antibiotics, Resistant cultivars, Vectors control/trap	Ferreira et al., 2021
16S-VA	Elm yellows group	Leaf chlorosis, witches broom, flower abortion, decline and die-back	Olive	Antibiotics, Resistant cultivars, Vectors control/trap	Pasquini et al., 2000
16S-XIIA	Stolbur group	Leaf chlorosis, witches broom, flower abortion, decline and die-back	Olive	Antibiotics, Resistant cultivars, Vectors control/trap	Pasquini et al., 2000
Oomycetes					
	*Phytophthora irregulare*	Root rot, basal stem cankers, defoliation, twig die-back	Field crop, citrus, olive	Chemical control, Biocontrol (antagonistic fungi and bacteria)	Drenth and Sendall, 2001; Santilli et al., 2020
Root rot	*Phytophthora megasperma*	Root rot, basal stem cankers, defoliation, twig die-back	Field crop, vegetables, cereals, olive	Chemical control, Biocontrol (antagonistic fungi and bacteria)	Drenth and Sendall, 2001; Santilli et al., 2020
	*Phytophthora oleae*	Root rot, basal stem cankers, defoliation, twig die-back	Olive	Chemical control, Biocontrol (antagonistic fungi and bacteria)	Ruano-Rosa et al., 2018
	*Phytophthora palmivora*	Root rot, basal stem cankers, defoliation, twig die-back	Palm, olive, Areca nut, fruit trees	Chemical control, Biocontrol (antagonistic fungi and bacteria)	Cacciola et al., 2007; Lucero et al., 2007; Lo Giudice et al., 2010; Chliyeh et al., 2013
	*Phytium schmitthenneri*	Root and crown rot	Oak, plum, chestnut, grapevine olive	Chemical control, Biocontrol (antagonistic bacteria)	Legrifi et al., 2022b, 2022a
	*Phytium speculum*	Root and crown rot	Oak, plum, chestnut, grapevine olive	Chemical control, Biocontrol (antagonistic bacteria)	González et al., 2016
Fungi					
	*Alternaria alternata*	Leaf necrosis, withering of leaf tips	Olive, apple, Asian pear		Lo Piccolo et al., 2014; Armitage et al., 2020
	*Arthrinium phaeospermum*	Leaf necrosis, withering of leaf tips	Olive		Lo Piccolo et al., 2014
Drupe rot Dalmatian disease Fruit rot	*Botryosphaeria dothidea*	Fruit necrotic lesions	Woody plant, fruit trees, shrub, olive	Chemical control	Nigro et al., 2018; Moral et al., 2019; Korukmez et al., 2020
White rot	*Botryosphaeriaceae* species	Branch dieback and necrosis	Blueberry, grapevine, palm, Eucalyptus, maple, oak, olive	Chemical control	Romero et al., 2005; Moral et al., 2010; Carlucci et al., 2013; Nigro et al., 2013
Olive anthracnose Soapy fruit	*Colletotrichum acutatum*, *C. boninense*, *C. gloeosporioides*	Fruit fallen, shoot and branch die-back	Fruit trees, olive	Chemical control, Resistant cultivars Early harvesting	González et al., 2006; Chattaoui et al., 2016; Moral et al., 2017b
	*Cytospora oleicola*	Twig and branch die-back	Olive	Chemical control	Úrbez-Torres et al., 2020
	*Cytospora olivarum*	Twig and branch die-back	Olive	Chemical control	Úrbez-Torres et al., 2020
	*Cytospora plurivora*	Twig and branch canker and die-back	Peach, plum, rosaceae, olive	Chemical control	Úrbez-Torres et al., 2020; Pan et al., 2020
	*Cytospora sorbicola*	Twig and branch canker and die-back	Rowan, olive	Chemical control	Úrbez-Torres et al., 2020; Pan et al., 2020
	*Diplodia seriata*	branch dieback and necrosis	Grapevine, maple, olive	Chemical control	Úrbez-Torres et al., 2013; Moral et al., 2017a
Fusarium wilt	*Fusarium oxysporum*	Cortical decay, root rot, leaf yellowing and wilting	Field crop, weeds, olive	Chemical control, Biocontrol (antagonistic fungi and bacteria)	El-Morsi et al., 2009; Ben Amira et al., 2017
	*Fusarium solani*	Cortical decay, root rot, leaf yellowing and wilting	Field crop, weeds, olive	Chemical Biocontrol, Biocontrol (antagonistic fungi and bacteria)	El-Morsi et al., 2009; Ben Amira et al., 2017
Charcoal-rot disease	*Macrophomina phaseolina*	Root rot, charcoal rot, basal stem rot	Field crop, cereals, olive	Chemical control	Sanei and Razavi, 2011; Moral et al., 2019
	*Neofusicoccum mediterraneum*	branch dieback and necrosis	Olive	Chemical control	Úrbez-Torres et al., 2020
Leprosy	*Phlyctema vagabunda*	Fruit necrotic lesions	Olive	Chemical control	Romero et al., 2018
	*Phoma cladoniicola*	Leaf necrosis, withering of leaf tips	Olive		Lo Piccolo et al., 2014
	*Pseudocercospora cladosporioides*	Leaf spot disease, defoliation	Olive	Chemical control, Resistant cultivars	Sergeeva et al., 2008; Avila and Trapero, 2010
Live leaf spot Scab Peacock spot	*Venturia oleaginea*	Leaf abscission	Olive	Chemical control, Resistant cultivars	Benítez et al., 2005; González-Domínguez et al., 2017
	*Pleurostoma richardsiae*	Trunk disease	Grapevine, olive	Chemical control	Carlucci et al., 2013; Nigro et al., 2013
	*Ulocladium consortiale*	Leaf necrosis, withering of leaf tips, twig dieback	Olive		Lo Piccolo et al., 2014
Verticillium wilt,	*Verticillium dahliae*	Wilting, chlorosis, foliar desiccation, xylem discoloration and die-back	Field crop, vegetables, weeds, olive	Tolerant cultivars, Soil solarization, Biostimulants	López-Escudero and Mercado-Blanco, 2011; Tsror, 2011; Montes-Osuna and Mercado-Blanco, 2020

*In most cases, an integrated disease/pest management (IPM) strategy is needed and highly recommended.

### 3.4 Bacteria

Olive knot disease caused by the Gram-negative phytopathogenic bacterium *P. savastanoi* pv. *savastanoi* is a relevant disease affecting olive trees globally, especially in the Mediterranean region (**Table 2**). It can cause severe damage in olive groves, producing serious losses in terms of production (Buonaurio et al., 2015; Moretti et al., 2016; Turco et al., 2022). This disease is characterized by hyperplasia (tumors, galls or knots) in aerial organs of the tree, mostly on stems and branches and, occasionally, on leaves and fruits (Ramos et al., 2012). Displaying chronic nature, the symptoms endure and reappear over years. *Pseudomonas savastanoi* infiltrates through wounds from harvesting, pruning, frost, hail, or leaf scars, initially colonizing nearby tissues, creating cavities with pectolytic and hemicellulolytic enzymes, or directly invading xylem vessels (Buonaurio et al., 2015; Košćak et al., 2023). Subsequently, bacterial virulence factors (i.e. indol-3-acetic acid and cytokinins) stimulate hyperplasia (Rodríguez-Moreno et al., 2008).

*Xylella fastidiosa* constitutes an emerging, major threat for olive trees. It is a xylem-limited gram-negative bacteria causing important economical losses in many crop, forest, and landscape plants (Saponari et al., 2014) (**Table 2**). It is the causal agent of the “olive quick decline syndrome” (OQDS) (Saponari et al., 2014). OQDS displays leaf scorching and twig desiccation, escalating in severity and spreading throughout the crown. Trees, irrespective of age, gradually decline and succumb (Saponari et al., 2019). The invasive CoDiRO strain of *X. fastidiosa* belongs to subspecies *pauca*, and it was probably introduced via infected ornamental plant material (Nigro et al., 2018). *Xylella fastidiosa* is transmitted by xylem fluid-feeding nearctic and neotropic sharpshooter leafhoppers (*Hemiptera, Cicadellidae*). In Europe, this group is limited, while spittlebugs (*Hemiptera, Cercopoidea*) are potential vectors. Therefore, to avoid the spread of the bacterium to other regions a disease management approach aimed at restraining bacterial dispersal by diminishing the inoculum sources and by targeting juveniles (mechanical weeding in late winter) and adults (a pesticide treatment in late spring when they move to olives) of the vector has been adopted (Corrado et al., 2016; Saponari et al., 2019).

Phytoplasmas, vector-borne and graft-transmissible bacteria, cause diverse plant diseases, and are classified into at least 37 16S rDNA groups (Wei and Zhao, 2022). Olive tree-infecting phytoplasmas are grouped as 16S-IB (Aster yellows group), 16S-VA (Elm yellows group) and 16S-XIIA (Stolbur group) (Pasquini et al., 2000) (**Table 2**). Recently, a new subgroup causing witches’ broom in olive trees has been found in Brasil (Ferreira et al., 2021). Affected trees exhibit a spectrum of symptoms: shoot proliferation, chlorosis and deformation of the leaves, bushy growth, witches’ brooms, flower abortion, bud failure, decline and die-back (Pasquini et al., 2000). Insights into olive phytoplasma epidemiology and spread remain limited. Developing or identifying resistant cultivars is a promising disease control strategy. While certain antibiotics might delay or ease symptom manifestation, this approach is not usually practical (Caglayan et al., 2011). The cicadellid *Hyalesthes* sp. is suspected of field-transmitting olive phytoplasma diseases. Physical prevention, such as screening or mineral coatings on plants, proves the most effective vector control method (Caglayan et al., 2011).

### 3.5 Oomycetes

The Oomycetes includes four orders, among which *Peronosporales* contains the well-known genera *Phytophthora* and *Pythium* (Drenth and Sendall, 2001) (**Table 2**). *Phytophthora* spp. cause 90% of the crown rots of woody plants (Lamour, 2013). It can infect the plant via roots and air, causing rot of roots and of the basal part of the stem in a wide range of hosts including olive (López-Escudero et al., 2008). Numerous members of this genus are recognized for inducing cause leaf chlorosis, wilting, defoliation and twig dieback in olive plants. They also contribute to root rot and basal stem cankers (Drenth and Sendall, 2001; Santilli et al., 2020). Soil moisture content influences pathogenicity of *P. megasperma, P. palmivora* and *P. irregulare*. These species have been reported to cause widespread root rot and rapid plant death only under continuous waterlogged soil conditions (Sánchez Hernández et al., 1998). *Phytophthora palmivora* has been reported as the etiological agent of root rot of fine roots and wilt of young olive trees both at nurseries and new plantings in Italy (Cacciola et al., 2007), Morocco (Chliyeh et al., 2013) and Argentina (Lucero et al., 2007). A synergy between this pathogen and *V. dahliae* has been reported as well (Lo Giudice et al., 2010). *Phytophthora oleae* was isolated from soils and roots of wild olive trees in protected natural areas of Southern Italy and Spain (Ruano-Rosa et al., 2018). Some species like *P. nicotianae* and *P. oleae* are occasionally adapted to an aerial lifestyle, infecting aboveground olive organs (e.g. drupes, leaves, and twigs) under moist environments thereby causing fruit rot, leaf dessication and twig dieback (Ruano-Rosa et al., 2018). Effective management of this pathogen mainly relies on the screening of olive cultivars resistant to *P. megasperma* and *P. inundata*, and on the use of fungicides and antagonistic fungi and bacteria (López-Escudero et al., 2008) (**Table 2**).

*Pythium* spp. have been characterized as causal agents of root rot in various woody species (**Table 2**). Attacks by this pathogen on cultivated olive trees have been reported in Spain (*P. speculum*) (González et al., 2016) and Morocco (*P. schmitthenneri*) (Legrifi et al., 2022b). Measures such as crop rotation, soil solarization, and mostly the use of fungicides, have been implemented to control these pathogens. Recently, effectiveness of *Alcaligenes faecalis* ACBC1 and *Bacillus amyloliquefaciens* SF14 to manage olive root rot caused by *P. schmitthenneri* was reported (Legrifi et al., 2022a).

### 3.6 Fungi

The olive tree can be attacked by a range of fungal pathogens which can infect different plant organs (leaves, flowers, fruits, roots and stems). Chliyeh and co-authors enumerated 124 olive fungal pathogens. Many of them (83 species) can be found in Europe, and their presence has been mostly reported in Italy (55 species), followed by Spain (46) and Greece (24) (Chliyeh et al., 2014).

#### 3.6.1 Fungal disease affecting leaves and fruits

The complex of fungal species *Colletotrichum acutatum sensu lato* (s. lat.), *C. boninense* s. lat., and *C. gloeosporioides* s. lat. causes olive anthracnose, the most critical and worldwide spread disease of olive drupes (Moral et al., 2017b) (**Table 2**). About 13 *Colletotrichum* species within this complex are known to impact this crop (Chattaoui et al., 2016). In addition to the premature fall of affected fruits, these pathogens also cause the dieback of shoots and branches through the production of phytotoxins in the rotten fruit (Moral et al., 2017b). While both inorganic and organic fungicides offer satisfactory control, effectiveness may show inconsistency under field conditions due to the tolerance displayed by the pathogen to copper, the key constituent of the primary fungicides used (Cacciola et al., 2012). Efficient and eco-friendly control measures involve early harvesting before full ripeness or selecting late maturing cultivars. Nonetheless, employing resistant cultivars stands as the most potent control method, which can complement biological, chemical, or cultural practices (Cacciola et al., 2012; Moral et al., 2017b).

A disease resembling olive anthracnose, referred as ‘drupe rot’ or ‘Dalmatian disease’, exhibits analogous symptoms (González et al., 2006). The classification of the causal agent has undergone multiple revisions. Research integrating genetic and morphological data attributed the olive fruit rot to *Botryosphaeria dothidea* (syn. *Camorosporium dalmaticum*) (Nigro et al., 2018). Symptoms of fruit rot include rounded, necrotic, depressed spots with well-defined borders. As the disease progresses the necrotic spots expand, eventually consuming the entire fruit (**Table 2**). This infection occurs in green fruit, typically in areas marked by *B. oleacea* stings. Consequently, a highly effective approach to manage drupe rot involves olive fly control through insecticide sprays, sometimes combined with copper-based fungicides.

*Venturia oleaginea* (Castagne) is another relevant pathogenic fungus affecting the aboveground part of the olive tree (Buonaurio et al., 2023). The taxonomy of this pathogen has changed several times (i.e. *Cycloconium oleaginum*, *Spilocaea oleagina*, *Fusicladium oleagineum*), but the genus *Venturia* is recommended to be used among pleomorphic genera in *Dothideomycetes* (Rossman et al., 2015). It induces the olive leaf spot (OLS), also known as ‘peacock’s eye’ disease or ‘scab’, leading to significant yield reduction in many olive-growing areas (**Table 2**). This fungus is a specific biotroph of the olive tree, causing leaf abscission and weakening of the whole tree (Benítez et al., 2005). OLS management encompasses an integrated strategy, with both pre- and post-planting measures. These include adequate practices, resistant cultivars, chemical applications, antagonistic microorganisms, natural antifungal products and plant resistance inducers (Buonaurio et al., 2023). Recently, the positively selected gene *evm.model.Chr16.1133*, identified in leaves of *O. europeae* subsp. *cuspidata*, has been related with major susceptibility to OLS (Wang et al., 2022).

*Pseudocercospora cladosporioides* (Sacc.) U. Braun (syn. *Cercospora cladosporioides, Mycocentrospora cladosporioides*) is another worldwide spread pathogen causing leaf spot disease in olive. It triggers severe defoliation when humid conditions persist during autumn and spring. Initial signs are light green patches on leaf surfaces, turning necrotic later on (**Table 2**). The distinctive black olivaceous fructifications of the fungus consistently cover the lower side of leaves (Pappas, 1993; Avila and Trapero, 2010). Petioles, fruit stalks and young shoots can be also affected (Pappas, 1993). This infection is easily confounded with OLS.

Leprosy of olive tree has been reported in different areas of Italy (Goidanich, 1964), Spain (Roca et al., 2007) and USA (Rooney-Latham et al., 2013). It affects fruits and is characterized by small dark rounded necrotic lesions surrounded by a chlorotic halo. It can also produce necrotic lesions on branches and leaves but with low frequency (Romero et al., 2018) (**Table 2**). The causal agent is *Phlyctema vagabunda* (Desm.), although the taxonomy has been revised several times. Leprosy symptoms have dramatically increased in recent years in southern Spain and Portugal, countries where the process of crop intensification is evident (Romero et al., 2018).

#### 3.6.2 Fungal pathogens involved in olive root rot

The most common fungi isolated from rotted roots of olive trees are *Fusarium oxysporum, F. solani, F. moniliforme, F. equiseti* and *Rhizoctonia solani*, while *Macrophomina phaseolina, Cylindrocarpon* sp.*, Acremonium egyptina, Chaetomium olivaceum* and *Nigrospora oryzae* are less frequently found (El-Morsi et al., 2009; Ben Amira et al., 2017). Obviously, the incidence of these species varies among locations (El-Morsi et al., 2009) (**Table 2**). *Fusarium* is considered a cosmopolitan soil saprophyte in the case of olive. However, under certain environmental conditions, some representatives of this genus become as facultative biotrophic parasites leading to cortical decay, root rot, leaf yellowing and wilting, and the untimely demise of the infected plant (Ben Amira et al., 2017). Death of plants at nurseries and of young trees in the field due to infection by *F. solani* has been described in Nepal (Vettraino et al., 2009) and Argentina (Pérez et al., 2011). Integrated management practices based on resistant cultivars, chemical, cultural and biological methods, and biotechnological approaches are being adopted for successful management of *Fusarium* diseases. Some positive results in the control of *Fusarium* spp. and *R. solani* have been reported with the use of antagonists such as *Trichoderma* spp. (Mousa et al., 2006; Ben Amira et al., 2017) and *Bacillus subtilis* (Jacobsen et al., 2004).

Another root pathogen causing serious economic losses in olive nurseries of Iran (Sanei and Razavi, 2011) and in olive orchards in South Spain (Moral et al., 2019) is *Macrophomina phaseolina* (Tassi), the causal agent of the charcoal-rot disease (**Table 2**). The development of charcoal rot seems to be favored by the combined effect of heat stress, soil-water deficit, light-textured soil and/or stress connected with the host reproduction (Sanei and Razavi, 2011). In contrast to the numerous pathogens which are favored by moist conditions, *M. phaseolina* becomes more problematic in agricultural areas where long drought periods and high temperatures prevail (Saleh et al., 2010). So far, only few cases of this disease have been found in olive trees (Sanei and Razavi, 2011).

#### 3.6.3 Fungi causing olive wilt and decline

Some fungal pathogens cause wilting, cankers, dieback and other decline-related symptoms in olive trees. Characteristic symptoms appear when water and nutrient demand surpass the conductive capacity of the vascular tissue. Verticillium wilt of olive (VWO), caused by the soil-borne hemibiotrophic fungus *Verticillium dahliae* Kleb. (subdivision *Deuteromycotina*, order *Hyphomycetes*) is considered one of the main limiting factors for olive cultivation, causing high tree mortality, productivity reduction, and lowered fruit yield (Montes-Osuna and Mercado-Blanco, 2020). The fungus penetrates the roots through micro- or macro-breakages, with occasional active penetration (Prieto et al., 2009; López-Escudero and Mercado-Blanco, 2011; Jiménez-Díaz et al., 2012). The subsequent invasion of the xylem vessels partially blocks the vascular system increasing resistance to water and nutrient flow within the plant (Pomar et al., 2004; Pascual et al., 2010). This may hamper water and nutrient transport to upper parts of the plant, causing the typical symptoms of a vascular disease: wilting, chlorosis, foliar desiccation and premature defoliation, xylem discoloration and plugging of vessels (Tsror, 2011). In the latter stages of the parasitic phase of its life cycle, the pathogen produces infectious propagules (microsclerotia) in dead or dying tissues of infected plants able to persist in the soil for a long time (Keykhasaber et al., 2018; Montes-Osuna and Mercado-Blanco, 2020). These dormant structures represent the main dispersal form of *V. dahliae* (López-Escudero and Mercado-Blanco, 2011). Severity of VWO hinges on the virulence of the infecting *V. dahliae* isolates, traditionally classified as defoliating (D) and non-defoliating (ND) pathotypes (López-Escudero and Mercado-Blanco, 2011). Symptoms are also influenced by soil inoculum density and host genotype (Bubici and Cirulli, 2012).

Efficient control of this disease must be achieved by implementing an integrated management strategy, with emphasis in preventive and sustainable measures (Rodríguez et al., 2008; López-Escudero and Mercado-Blanco, 2011; Tsror, 2011; Jiménez-Díaz et al., 2012; Keykhasaber et al., 2018; Montes-Osuna and Mercado-Blanco, 2020). Concerning preventive control measures, the use of tolerant/resistant varieties is the main cost-efficient and long-lasting means to handle VWO (Markakis et al., 2022; Serrano et al., 2023). Some rootstocks and commercial cultivars of olives have demonstrated delayed disease onset, capacity for recovery, and minimal plant losses under *V. dahliae* pressure. Regrettably, these are not commonly adopted in commercial olive cultivation due to their limited agronomic qualities (for more information see López-Escudero et al., 2004; Martos-Moreno et al., 2006; Bubici and Cirulli, 2012; García-Ruiz et al., 2015; Sanei and Razavi, 2017; Serrano et al., 2023). The use of non-infested soils, and the early and reliable *in planta* (Mercado-Blanco et al., 2002; Mercado-Blanco et al., 2003; Karajeh and Masoud, 2006; Markakis et al., 2009; Gramaje et al., 2013; Aslani et al., 2017) and in soil (Pérez-Artés et al., 2005; Bilodeau et al., 2012; Moradi et al., 2014; Ju et al., 2020) detection of the pathogen is also crucial for the effective management of VWO.

Regarding measures after planting, effective control of disease incidence and severity involves the proper irrigation system management (Baroudy et al., 2018), the adoption of cultivation practices preventing root damage and the use of decontaminated equipment (Tsror, 2011). The utilization of thermal treatments such as soil solarisation (Tjamos and Jiménez-Díaz, 1998) or hot air (Morello et al., 2016) on established orchards was slightly efficacious. The use of endo-therapy, a technique consisting in the direct delivery of active compounds into the plant vascular system by physical injections has been recently suggested (Grandi et al., 2023). Beneficial endophytes are promising candidates for biocontrol against *V. dahliae*, in particular the arbuscular mycorrhizal fungi (AMF) (Castillo et al., 2006; Porras-Soriano et al., 2006; Chatzistathis et al., 2013; Aleandri et al., 2015; Carrero-Carrón et al., 2016; Ruano-Rosa et al., 2016; Boutaj et al., 2019). Bacteria isolated from olive plants also showed antagonistic activity against *V. dahliae*, in particular members of the genera *Bacillus* and *Pseudomonas* (see section 5).

Other fungi causing olive tree decline are worth mentioning. For instance, olive cankers and resulting dieback associated with the fungus *Phoma incompta* (Sacc. & Mart.) were earlier reported in Greece (Malathrakis, 1979). Also in Greece, *Cytospora oleina* (Berl.) and *Eutypa lata* (Pers.: Fr.) were characterized as the causal agents of olive branch dieback (Rumbos, 1988). *Botryosphaeria dothidea* (Moug.: Fer.), *Diplodia seriata* (de Not.), and *Neofusicoccum mediterraneum* (Crous) were described to cause branch dieback and necrosis, blight, and subsequent death of olive shoots in Spain, Italy and California (Úrbez-Torres et al., 2013; Moral et al., 2017a) (**Table 2**). Úrbez-Torres and co-authors identified 18 fungal species causing olive twig and branch dieback in California (USA), *Botryosphaeriaceae* being the most prevalent followed by species of *Diaporthe* and of *Diatrypaceae.* Likewise, in USA, *C. oleicola* (D.P. Lawr., L.A. Holland & Trouillas), *C. olivarum* (Úrbez-Torr., D.P. Lawr., Peduto, Gubler & Trouillas), *C. plurivora* (D.P. Lawr., L.A. Holland & Trouillas), and *C. sorbicola* (Norphanphoun, Bulgakov, T.C. Wen & K.D. Hyde) were associated with olive tree branch cankers and dieback (Úrbez-Torres et al., 2020) (**Table 2**). Upon inoculating olive trees, all the species mentioned above induced varying-sized lesions, with *N. mediterraneum* generating the largest ones, followed by *Diplodia mutila* (Fr.) (Úrbez-Torres et al., 2013). In Italy, *Pleurostoma richardsiae* (Nannfeldt), *Phaeoacremonium* spp., and members of *Botryosphaeriaceae* have emerged as predominant fungi associated with olive decline (Carlucci et al., 2013; Nigro et al., 2013) (**Table 2**). Other fungi have also been found at lower incidence or just incidentally. Nevertheless, they have been shown to cause dieback and decline-related symptoms in olive trees. Among these fungi, *Diaporthe foeniculina* (Sacc.) (Úrbez-Torres et al., 2013; Moral et al., 2017b), *Diaporthe rudis* (Fr.) Nitschke, *Diatrype oregonensis* (Whem), *Diatrype stigma* (Hoffm.) Fr., *Ilyonectria destructans* (Zinssm.), *Comoclathris incompta* (Sacc. & Mart.) (reported as *Phoma incompta*), and members of the *Basidiomycota*, such as *Fomitiporia mediterranea* (M. Fish.), *Schizophyllum commune* (Fr.:Fr.) and *Trametes versicolor* (L.:Fr.) can be cited (Rumbos, 1993; Úrbez-Torres et al., 2013; Carlucci et al., 2013; Moral et al., 2017a).

## 4 Olive roots and belowground microbiota: who shapes whom in the holobiont?

Plant roots are able to select soil-inhabiting microorganisms, and this “recruitment” influences the microbiome composition of the different compartments of this organ (i.e. rhizosphere, rhizoplane and endosphere) (Edwards et al., 2015). The so-called concept of plant “cry for help” refers to the phenomenon by which plants release chemical signals in response to stress or damage, thereby attracting beneficial organisms or triggering defense mechanisms (Rizaludin et al., 2021). These signals, mostly volatile organic compounds (VOCs) (Baldwin et al., 2006) and root axudates (Tiziani et al., 2022), can serve as a communication system between the plant and its environment, enabling the plant to enhance its own survival and fitness.

Recent works suggested that root’s architecture and morphology also play a role in the assembly and functioning of the rhizosphere microbiome (Herms et al., 2022). Traits associated with root architecture, such as root length, biomass and branching density, possess the potential to exert influence on the rhizosphere microbiome by impacting the root system as a whole (Herms et al., 2022). Moreover, based on genome-wide association studies (GWAS) it has been demonstrated that the plant associated microbiota is sensitive to the host genotype. The association between host loci and the abundance of a specific subset of rhizosphere microorganisms was found in *Arabidopsis thaliana* (Bergelson et al., 2018) and Sorghum bicolor (Deng et al., 2021). These works showed that the root microbiome was shapped by specific loci related with immune defence and root structure and physiology (e.g. cell wall integrity, root and root-hair development). On the other hand, the belowground microbiota can also influence root traits. Several investigations have unveiled the ability of some PGPR to alter root architecture and morphology by releasing auxins and cytokinins (Kudoyarova et al., 2017). More than seventy bacterial strains including a wide range of phyla able to altering plant root growth have been recently examined in this regard (Grover et al., 2021).

Regarding olive very few studies are available about root structure. Tan and co-authors found a positive relation between the increase of salt concentration and the intensification of root structural plasticity. With the increase of salinity, the plant favoured the production of thicker roots with bigger diameters that could better protect the stele from water loss (Tan et al., 2020). Our own studies have unveiled the existence of a strong relation between specific root traits and tolerance/susceptibility to VWO. Indeed, *V. dahliae*-susceptible olive varieties present root systems with higher plasticity and lateral development compared with the tolerant ones (Cardoni et al., 2021; 2022). We have also detected differences in the taxonomical composition of the olive root-associated microbiota depending on the tolerance level towards VWO of the cultivar examined. Thus, the rhizosphere of a *V. dahliae*-tolerant variety presented a higher relative abundance of genera often described as PGPM while the susceptible cultivar showed higher prevalence, in this case in the root endosphere compartment, of fungal genera well known for including phytopathogenic species (Fernández-González et al., 2020a). From these findings the presence of a relationship between the architecture (and composition) of the olive root system and its associated microbial community could be argued. For instance, previous work showed that a root system with low branch root orders, as found for VWO-tolerant olive cultivars (Cardoni et al., 2021), is a hotspot for PGPB selection (Wang et al., 2019), beneficial microorganisms being reported to be in higher abundance in *V. dahliae*-tolerant olive varieties (Fernández-González et al., 2020a). Many other root functional traits can contribute to shape the structure and composition of the olive root-associated microbial community. For example, larger or smaller contact surfaces between the soil and the root could influence the spatial differentiation of the root-associated microbiota, due to the higher or lower availability of different trophic niches to be colonized by soil-borne microorganisms. Likewise, the quantity of soluble carbon depositions, related to the number of root tips, could affect the structure and abundance of rhizosphere and endosphere communities, for example through the modification of soil pH and moisture. In the same way, qualitative and quantitative differences of specific components of the olive root-associated microbiota may in turn influence the development of different typologies of root systems. Indeed, numerous PGPR, assume significant roles in modulating root architecture and growth through the secretion of phytohormones, volatile organic compounds (VOCs), and secondary metabolites (Grover et al., 2021). For example, a recent study showed that the antagonistic ability of the BCA *P. simiae* PICF7 against *V. dahliae* may rely, at least partially, on the production of a repertory of VOCs, some of them with plant growth promoting activities (Desrut et al., 2021; Montes-Osuna et al., 2022). Another aspect that should be considered in the relation between olive root traits and the associated microbiome is the biochemical composition of the roots. A recent work demonstrated the presence of significant differences in the composition of the basal secondary metabolic profile of the roots of VWO-tolerant and VWO-susceptible olive cultivars. The first ones showed a higher amount of secoiridoids, family of compounds that displayed *in vitro* microbial activity against bacteria fungi (Cardoni et al., 2023b). The presence of these compounds in root tissues could be a selection factor in shaping the root-associated microbiota of tolerant olive cultivars, especially in the root endosphere. However, studies on the relation between structural and biochemical traits of olive roots and the composition of the associated microbiota are yet to be conducted.

## 5 Unveiling the olive microbiome and factors influencing its composition, structure and functionality

**Figure 3:**
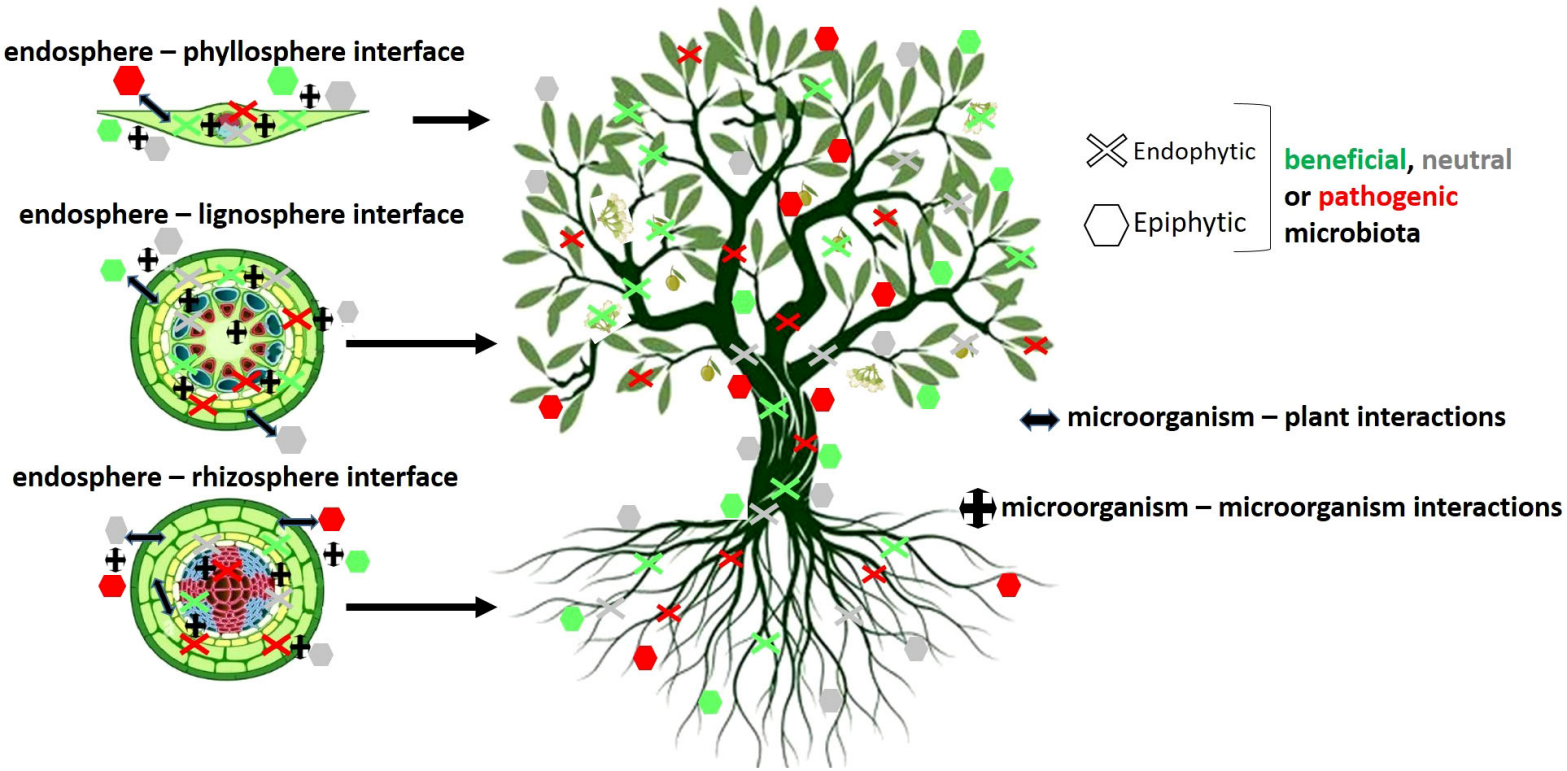
Schematic representation of the interactions between the olive plant and its associated microbial communities present in above- and belowground compartments. Based on Bettenfeld et al. (2020).

Plants do not live alone but in close association with complex microbial communities. The complete assemblage of microorganisms residing on or within a plant constitutes the plant microbiota, while the collective genetic material of these microorganisms defines the plant microbiome. The dynamic interactions between the host and its microbial inhabitants, as well as among the constituents of the resident microbiota, are essential for the health, fitness, adaptation and resilience of the so-called plant holobiont (Hardoim et al., 2015; Berg et al., 2016). This meta-organism can be defined as ‘*the genomic reflection of the complex network of symbiotic interactions that link an individual of a given taxon with its associated microbiome*’ (Bettenfeld et al., 2020) (**Figure 3**). The co-evolution of microbes and their hosts results in intimate relationships that create specific and stable microbiomes. Recently, Petipas and colleagues introduced the concept of microbe-mediated adaptation in plants, which is defined as an improvement in the host´s fitness within a specific environment, partly or entirely attributable to interactions with microorganisms (Petipas et al., 2021). Defining the core microbiome of a given plant holobiont is essential to better understand its contribution to the health and productivity of the host, to shade light on species-specific relationships, and to increase the efficiency of bioinoculants (Santoyo, 2022). Besides, the composition of the plant-associated microbiome is influenced by several biotic and abiotic factors that can influence its structure and functionality. These factors can be climatic and environmental changes, pesticide treatments, soil type and structure, plant health, plant developmental stage, and/or infection by pathogens and pests (reviewed by Berg and Smalla, 2009).

Regarding the olive holobiont a growing number of studies is available on the description of its associated microbiome. Nevertheless, they are mostly focused on specific plant compartments/organs (e.g. fruit, root endosphere, rhizosphere, xylem, etc.) and/or a particular influencing factor (e.g. olive genotype, cropping system, tree phenology, tolerance/susceptibility to a disease, etc.) (**Figure 3**, **Table 3**). Concerning the aboveground part of the tree, Pascazio and co-authors described alterations in bacterial community composition between the olive phyllosphere (leaf) and carposphere (fruit) (**Table 3**). Furthermore, they showed that the adoption of sustainable soil management practices enhanced the number of bacterial species present in the fruit (Pascazio et al., 2015). The impact of the olive genotype on the bacterial communities present in fruit, leaf and soil has also been described (Malacrinò et al., 2022) (**Table 3**). This work showed that the genotype exerted varying impacts on the diversity, structure, composition, and co-occurrence network within each plant compartment, with a more pronounced influence observed in fruits as opposed to leaves or soil. This study confirmed the results of a previous work about the characterization of the bacterial and archaeal community inhabiting the leaves of different olive genotypes originating from diverse Mediterranean areas (Müller et al., 2015) (**Table 3**). These authors concluded that the influence of the genotype was stronger than that of soil and climate conditions. A complete description of the olive leaf endophytic bacterial community subjected to different management (traditional *vs.* intensive cultivation) and saline stress has been recently published (Vita et al., 2022) (**Table 3**). A significant change in the resident microbiota was reported for plants exposed to moderate salt stress, while no modifications were observed under extreme salt-stress conditions. The changes were related with a shift of the bacterial community towards specific bacterial taxa able to survive in an environment enriched in salt, such as *Burkholderia* and *Ralstonia* (Vita et al., 2022). The composition of the fungal community present in the olive phyllosphere and carposphere was earlier reported (Abdelfattah et al., 2015) (**Table 3**). Results showed higher fungal diversity in leaves compared with that in flowers and fruits, and major significant changes in the latter organ. These authors deduced that the fungal community in fruitlets closely resembled that of the originating organs (flowers) and underwent progressive evolution within the fruit community. Furthermore, ripe fruits showed different species belonging to pathogenic and beneficial fungal genera (**Table 3**). A similar consortium was also found when analyzing culturable fungal epiphytes and endophytes present in fruits of two cultivars differing in susceptibility to anthracnose (**Table 3**). Furthermore, the host genotype was key in determining the endophytic but not the epiphytic fungal communities. However, a clear link between resistance to anthracnose and the identified fungal endophytes could not be established (Preto et al., 2017). Recently, the epiphytic fungal communities present in olive cultivars displaying different tolerance to anthracnose have been described as well (Castro et al., 2022) (**Table 3**). In this study neither the cropping system nor the plant genotype significantly influenced the composition of the fungal community, while the fruit maturation stage produced a strong impact on the epiphytic mycobiome. Furthermore, a significant correlation between the presence of *Saccharomycetales* spp. and lower susceptibility to anthracnose was reported. Association between the olive leaf microbial community and disease resistance was also found for *X. fastidiosa* (Vergine et al., 2020). Thus, the susceptible cv. Cellina di Nardò showed a drastic dysbiosis after *X. fastidiosa* infection, while the tolerant cv. Leccino maintained a similar microbiota both in infected and uninfected plants. Furthermore, the diversity of the microbiota in non-inoculated tolerant plants was greater than that in susceptible control plants. Interestingly, multiple bacterial taxa specifically associated with the resistant cultivar displayed interaction with *X. fastidiosa* (**Table 3**).

**Table 3:** Main information available about olive-associated microbial communities.

Olive cultivar	Organ/ compartment	Analysis or method implemented	Stress/external factor	Studied phyla	More abundant taxa/Core composition	References
Aboveground part						
Maiatica	Leaf and fruit	16S rRNA fingerprinting by PCR-DGGE	Soil management systems	Bacteria	*Rahnela*, *Enterococcus* and *Kluyvera* in the fruit pulp	Pascazio et al., 2015
Sinopolese and Ottobratica	Leaf, fruit, soil	Illumina amplicon sequencing	Plant genotype	Bacteria	*Pantea and Pseudomonas* in the fruit*, Escherichia-Shigella* and *Mucilaginobacter* in the leaf and *Sphingomonas* and *Acinetobacter* in the soil	Malacrinò et al., 2022
Arbequino, Ocal, Leccino, Koroneki, Kalamata, Chétoni, Trylia, Picholine, Maroccaine, Galega, Aglandau, 9 wild genotypes	Leaf	Illumina amplicon sequencing	Plant genotype	Bacteria and Archea	*Pelomonas, Ralstonia, Nitrososphera* and *Pseudomonas*	Müller et al., 2015
Frantoio, Lecciana, Leccino and Oliana	Leaf	Illumina amplicon sequencing	Saline stress	Bacteria	*Burkholderiales, Enterobacterales* and *Pseudomonadales*	Vita et al., 2022
Ottobratica	Leaf, flower, fertilized fruitlets and fruit	454 sequencing	Phenological stage	Fungi	*Aureobasidium, Colletotrichum, Pseudocercospora, Cladosporium, Devriesia* *Fusarium, Neofusicoccum, Alternaria, Cladosporium* and *Aureobasidium*	Abdelfattah et al., 2015
Verdeal-Transmontana and Madural	Fruit	DNA sequencing	Anthracnose	Fungi	*Cladosporium, Biscogniauxia* and *Alternaria*	Preto et al., 2017
Cobrançosa and Madural	Fruit	Illumina amplicon sequencing	Olive orchard management system and olive maturation stage	Fungi	*Saccharomycetales, Dothideales, Trichosporonales* and *Mucorales*	Castro et al., 2022
Leccino and Cellina di Nardò	Leaf	Illumina amplicon sequencing	Olive quick decline syndrome	Bacteria and Fungi	*Pseudomonas, Ammoniphilus* and *Limnobacter*	Vergine et al., 2020
Koroneki and Chondrolia Chalkidikis	Leaf, fruit, flower, root and soil	Illumina amplicon sequencing	Annual developmental cycle	Bacteria and Fungi	*Acinetobacter, Cutibacterium and Aureobasidium pollulans, Clodosporium* (aboveground part), *Streptomyces, Steroidobacter* and *Dactylonectria, Chrysosporium* (belowground part)	Kakagianni et al., 2023
Cobrançosa, Galega vulgar, Madural, Picual, Verdeal Transmontana	Leaf and twing	Illumina amplicon sequencing	Plant genotype	Fungi	*Pleosporales, Chaetothyriales, Myriangiales* and *Helotiales*	Costa et al., 2021
Cobrançosa	Leaf, twing and root	DNA sequencing	Season and location	Fungi	*Phomopsis, Fusarium, Trichoderma* and *Macrophomina*	Martins et al., 2016
Xylem						
Picual, Acebuche and Arbequina	Xylem sap	Illumina amplicon sequencing	Sap extraction methods	Bacteria and Fungi	*Acidibacter, Curtobacterium* and *Cutibacterium*	Anguita-Maeso et al., 2020
Picual and Arbequina	Xylem sap	Illumina amplicon sequencing	Plant age and genotype	Bacteria and Fungi	*Anoxybacillus, Cutibacterium*, and *Bradyrhizobium*	Anguita-Maeso et al., 2021b
Ac 18, Ac 15 and Picual	Xylem sap	Illumina amplicon sequencing	Verticillium wilt	Bacteria and Fungi	*Acidibacter, Anoxybacillus, Bradyrhizobium* and *Corynebacterium*	Anguita Maeso et al., 2021b
Kalamata and FS17	Xylem tissue	Illumina amplicon sequencing	Xylella fastidiosa and season	Bacteria and Fungi	*Methylobacterium, Sphingomonas, Pseudomonas* and *Kabatiella, Pyrenochaeta, Neococurbitaria*	Giampetruzzi et al., 2020
Belowground part						
	Soil and root	fluorescent terminal restriction fragment length polymorphism (FT-RFLP) analysis	Plant genotype and geography	Bacteria		Aranda et al., 2011
Acebuche, Lechín, Manzanillo, Picudo, Picual, Nevadillo	Soil and root	Pyrosequencing and FT-RFLP	Edaphic, climatic and Agronomic factors	Nitrifying Bacteria and Archaea	*Nitromonas, Nitrospira* and *Nitrobacter*	Cáliz et al., 2015
Frantoio	Root	PCR-denaturating gradient gel electrophoresis (PCR-DGGE)	Olive orchard management system	AMF community	*Glomus* *Macrocarpum, Sclerocystis sinuosa* and *Sclerocystis*	Palla et al., 2020
	Soil	Culture-dependent methods	Olive orchard management system	Bacteria and Fungi		Sofo et al., 2014
Frantoio and Leccino	Root	Illumina amplicon sequencing	Plant genotype and season	Bacteria and Fungi	*Proteobacteria, Actinobacteria* and *Agaricomycetes, Glomeromycetes*	Chialva et al., 2021
36 different varieties	Root	Illumina amplicon sequencing	Plant genotype	Bacteria and Fungi	*Gammaproteobacteria, Actinobacteria*, candidate división *WPS1* and *Mucoromycetes, Eurotiomycetes, Sordariomycetes*	Fernández-González et al., 2019
Frantoio and Picual	Soil	Illumina amplicon sequencing	Olive orchard management system	Bacteria and Fungi	*Gp6, Gp4*, candidate división *WPS1, Gemmatimonas, Sphingomonas* and *Solicoccozyma, Glomus, Fusarium, Mortierella*	Fernández-González et al., 2020b
Picual	Leaf and root	Illumina amplicon sequencing from Jiménez-Ruiz et al., 2017	Verticillium wilt	Bacteria and Fungi	*Saccamoeba lacustris, Sterkiella histriomoscorum, Cryptodifflugia operculata, Rhizoctonia solani*	Martí et al., 2020
Frantoio and Picual	Root	Illumina amplicon sequencing	Verticillium wilt	Bacteria and Fungi	*Gp6, Gp4, Streptimyces, candidatus Saccharibacteria* and *Rhizophagus, Olyonectria, Corynespora, Spizellomyces*	Fernández-González et al., 2020a

The bacterial and fungal communities associated with the above- and belowground parts of two olive cultivars, and their changes during the annual developmental cycle, have been recently described in detail (Kakagianni et al., 2023) (**Table 3**). Results showed that the microbiome was more influenced by the tree phenology than by the genotype, and that fungal and bacterial communities were more similar in the aerial plant parts than in the roots. Huge differences in the composition of the endophytic fungal communities of leaves and twigs were also found among five different varieties (Costa et al., 2021) (**Table 3**). In this study, the genotype explained about 30% of the fungal community variation, while the plant organ explains only 10% of the mycobiome variance. Moreover, the fungal community of twigs showed higher variation among cultivars compared with that present in leaves. Previously, the effect of the season and geographic location on the structure of the fungal endophytic communities of different olive organs was also reported (Martins et al., 2016) (**Table 3**). These authors found that mycobiome diversity and abundance were higher in roots compared with fungal communities present in leaves and twigs.

Few studies are available on the microbiota associated with the olive xylem vessels. Anguita-Maeso and collaborators described the chemical and microbial composition of the xylem sap extracted from stems of different cultivars (**Table 3**). They suggested that differences found between genotypes, mainly in the mycobiota composition, could be related with their different level of resistance against vascular pathogens (Anguita-Maeso et al., 2021a). Previously, the same research team reported the influence of the olive genotype on the composition of the endophytic bacterial community present in stems (Anguita-Maeso et al., 2020) (**Table 3**). In this study, ten keystone bacterial genera were proposed as the olive xylem core microbiome. Moreover, these authors speculated that this core could be vertically transmitted from one generation to the next one, and that its constituents may provide benefits such as plant growth promotion and antagonism towards *V. dahliae* (Anguita-Maeso et al., 2021b). Contrariwise, no correlation was found between the endophytic communities present in the olive xylem tissue and their different level of resistance towards *X. fastidiosa* (Giampetruzzi et al., 2020) (**Table 3**). Indeed, host genotype had a minor effect on the community composition and no clear association of specific taxa with the resistance of the cultivar was found.

Regarding the belowground microbiota Aranda and co-authors early described the bacterial communities present in the rhizosphere and root endosphere of wild olive genotypes in different Spanish locations (**Table 3**). In their work, 94 bacterial species with antagonistic activity against *V. dahliae* were isolated, showing that the root endosphere constitutes an important reservoir of bacteria with antagonistic potential against this pathogen (Aranda et al., 2011) (see section 5.3). A number of studies highlight the effects caused by cropping systems and soil management practices on the olive belowground microbiota, or on some of its constituents. These impacts have been recently reviewed (Melloni and Cardoso, 2023). For example, nitrifying microorganisms present in the rhizosphere of different olive cultivars, and the influence of edaphic, climatic and agronomic factors were examined by Cáliz and co-coworkers (Cáliz et al., 2015) (**Table 3**). This study revealed that ammonia-oxidizing archaea were more influenced by soil texture and olive varieties while the bacterial community was more affected by the soil management system. The strong influence of agronomic practices on the diversity and composition of specific components of the olive root microbiota has been studied as well (Palla et al., 2020) (**Table 3**). Earlier, the medium-term effects (12 years) of ‘sustainable’ *vs.* ‘conventional’ cropping systems on soil microbial composition and metabolic diversity in an olive orchard were investigated (Sofo et al., 2014) (**Table 3**). This study demonstrated the positive effect that a sustainable management has on both the soil microbiota composition and functionality, mainly through the periodic application of organic matter. Tree age and adaptation to climatic conditions can also influence the olive belowground microbiota, and its stability can have important consequences for the holobiont. For instance, the root endosphere microbiota of cold-resistant and cold-sensitive varieties were examined during spring and winter seasons (**Table 3**). Interestingly, the microbial community of older trees (> 20 years) was more stable than the younger ones, with low variations across seasons and genotypes. Only fine seasonal-dependent community modifications in the cold-susceptible genotype, which involved beneficial microbes and pathogens, were detected (Chialva et al., 2021).

The first in-depth characterization of the composition and structure of the olive root-associated microbiota has been accomplished only recently (Fernández-González et al., 2019) (**Table 3**). Indeed, a collection of different cultivars present in the World Olive Germplasm Collection (Córdoba, Spain), growing under the same agronomic, climatic, and pedological conditions, was analyzed to best represent the olive genetic diversity within the Mediterranean Basin. It was demonstrated that the endophytic and rhizosphere microbial communities are mainly shaped by the olive genotype. Furthermore, this factor was more determinant for the rhizosphere than for the endosphere, and more important for bacteria than for fungi. The impact of external factors on the olive rhizosphere was further confirmed by comparing holm oak (*Quercus ilex* L.) and olive tree belowground microbial communities subjected to different soil management (Fernández-González et al., 2020b) (**Table 3**). An important conclusion of this study was that intensive soil management practices influenced the rhizosphere communities to a much larger extent than the cultivar/genotype. Moreover, no correlation between the composition and structure of the rhizosphere microbial community and tolerance/susceptibility to *V. dahliae* of the olive cultivars was found. This result was confirmed by examining the impact that the inoculation with *V. dahliae* had on the rhizosphere and root endosphere microbial communities of two olive cultivars differing in VWO tolerance/susceptibility. Indeed, the belowground microbial communities of both cultivars displayed similarities, and the introduction of the pathogen did not induce noteworthy changes in their structure or functionality. The presence of *V. dahliae* caused significant differences only in the topology of the co-occurrence networks. This study suggested a link between the modification in the microbial community network, especially in the endosphere, and VWO susceptibility/tolerance (Fernández-González et al., 2020a). This result is in accordance with the conclusion reached by another study reporting the dynamics of the olive root microbiome subjected to perturbations such as *V. dahliae* infection and root mechanical damage (Martí et al., 2020) (**Table 3**). According to these authors, the onset of VWO is the consequence of a complex process involving more contenders than just the host plant and *V. dahliae*. Finally, the combined used of different omics approaches made it possible to study the interaction between the olive belowground microbiome and the host transcriptome, providing a holistic approach within the holobiont perspective (Fernández-González et al., 2021) (**Table 3**). This study aimed to classify olive genes and microbial operational taxonomical units (OTUs) that might play a pivotal role in the olive holobiont’s adaptation to particular natural conditions. For example, a positive correlation was found between two thaumatin-like coding genes, which are linked to plant defense via apoplastic antifungal activity, and several genes in cell wall synthesis or strength and the relative abundance of *Actinophytocola* OTUs. (Fernández-González et al., 2021).

To end this section, we would like to briefly mention the potential influence that rootstocks may exert on the olive microbiome, a scenario which has not been investigated for this holobiont. Similarly, to other tree crops olive can be cultivated by grafting a scion within a rootstock (Warschefsky et al., 2016, and references therein) to enhance plant production and/or tolerance to stresses (see, for instance, Díaz-Rueda et al., 2022, and references therein). As proved for grapes or apples, among other crops, rootstocks can act as “filters” to attract and select components of the soil microbiome, thereby “recruiting” microorganisms that provide benefis to the plant host (Marasco et al., 2018). Moreover, this process can be finely tuned when testing scion/rootstock combinations (Vink et al., 2021; Marasco et al., 2022).

### 5.1 Interactions among components of the olive microbiome

Microorganisms of the plant holobiont do not only interact with the host, but also among them creating complex networks through various types of interactions. These interactions can be antagonistic, such as competition for a limiting resource (e.g. biosynthesis of siderophores) or direct interference (e.g. production of antibiotics), or cooperative (e.g. quorum sensing) (Berry and Widder, 2014) (**Figure 3**). The functional capacity of the plant microbiome goes beyond the mere aggregation of its individual constituents, since they often engage in robust and frequent interactions. The relationships among microbial taxa and between them and the host can be likened to a delicate equilibrium. Besides, disturbing this balance can lead to adverse consequences for the host’s health (Barberán et al., 2012; Buonaurio et al., 2015). A valuable strategy for enhancing our understanding of the potential interactions within a specific microbial community involves the in-depth scrutiny of co-occurrence networks. These networks are typically formed by assessing correlations among the abundances of individual entities (e.g. microbial taxa) (Barberán et al., 2012). This kind of analysis can provide a more precise approach to identify patterns that may be more problematic to detect than by using the standard diversity metrics (e.g. alpha and beta) broadly used in microbial ecology (Barberán et al., 2012). It has been demonstrated that network’s complexity positively related with higher productivity of the host [e.g. in maize (Jiemeng et al., 2018)] or with its ability to suppress disease [e.g. in tobacco (Yang et al., 2017)].

The analysis of the co-occurrence network of the olive associated communities could help to shed light on the specific interactions taking place in the olive holobiont to respond to an external stress, such as pathogen attack. For example, the study of the interactions among components of the endophytic microbial community inhabiting the leaves of olive plants infected by *X. fastidiosa* showed that, in the presence of the pathogen, keystone species and the ratio between positive and negative network interactions were very different to those observed in healthy leaves. Indeed, some bacterial taxa were only found in the presence of the pathogen (Vergine et al., 2020). Similar results were reported when studying co-occurrence interactions in the root endosphere and rhizosphere communities of two olive cultivars differing in tolerance to VWO (Fernández-González et al., 2021). The endosphere community network showed the most significant alteration after *V. dahliae* inoculation, including changes in network parameters and keystone species. Likewise, changes in the microbial community co-occurrence network can occur not only as a consequence of the introduction of a deleterious microorganism, but also upon the inoculation with beneficial rhizobacteria able to confront it. This has been recently demonstrated in the case of two effective BCAs against *V. dahliae* (i.e. *P. simiae* PICF7 and *Paenibacillus polymyxa* PIC73), either in the presence or absence of the pathogen (Cardoni et al., 2023a). It has been suggested that the distinctive changes in network’s topology provoked by the introduction of these BCAs could explained, at least to some extent, different biocontrol strategies that are equally effective. Furthermore, recent results showed that drought also regulated the co-occurrence interactions among olive rhizobacteria by determining specific modules enriched with the so-called ‘aridity winners’ (see above), which included bacteria with multiple PGP functions against dryness (Marasco et al., 2021). These studies are good examples of the potential that these combined omics/bioinformatic approaches have to unveil the functioning of the olive holobiont under different scenarios.

### 5.2 Abiotic and biotic disturbances affecting the microbiome of the olive holobiont

Several studies have highlighted the support that the plant-associated microbial community provides to the host to mitigate biotic and abiotic stresses. The consequences of these stresses on the microbial component of a given plant holobiont are otherwise less explored. Indeed, the composition and structure of the plant-associated microbiome undergo alterations along time and space due a wide range of factors (Mercado-Blanco et al., 2018). Under natural conditions, plants and their microbiomes encounter a wide range of environmental conditions, including fluctuations in temperature, humidity, pH and exposure to UV rays, which directly or indirectly influence their composition (Santos and Olivares, 2021). Rain fall, and in general water availability, is also a relevant climatic factor influencing the biomass, activity, and composition of bacterial (Landesman and Dighton, 2010; Felsmann et al., 2015) and fungal (Rasmussen et al., 2020) communities of different plant species. Also heat disturbance, such as those caused by wildfire strongly influence the plant-associated microbiome, increasing the environmental selection pressure (van der Voort et al., 2016) and causing a slowdown of microbial processes linked to soil C and N dynamics (Yang et al., 2020). Anthropogenic factors, such as pollution produced by industrial and mining activities can also shape the microbiome of tree crops (Janus et al., 2005). Agricultural practices have a strong impact on the plant microbiome through alterations in soil properties, mainly nutritional. These practices can impact the microbiome directly by either stimulating or inhibiting its activity based on the nutritional preferences of microorganisms, or indirectly by influencing how plants “recruit” their microorganisms. (Santos and Olivares, 2021).

Plant pathogens are probably the most important external biotic factor influencing the plant-associated microbiota. In a scenario of pathogen invasion, the plant-associated microbial communities are able to modify their activity and functional responses to stimulate host’s health and defence responses (Byers et al., 2020). On the other hand, pathogens can occupy the ecological niche where other microorganisms live, causing competition for space and resources (e.g. nutrients) and a shift in the indigenous microbial community (Mawarda et al., 2022). Finally, inoculation with beneficial microorganisms may also shift the indigenous plant-associated microbiome. Indeed, beneficial bacteria, even if originating from plant-associated microenvironments, may perturb the indigenous microbiome if applied to plant roots in sufficient numbers (Scherwinski et al., 2008; Erlacher et al., 2014; Eltlbany et al., 2019).

Regarding olive, the information available on abiotic factors that can shape its associated microbiome primarily concentrates on the impact of agronomic practices (**Table 3**). Likewise, the influence of weather conditions on leaf, fruit and root microbiomes has been examined (Cáliz et al., 2015; Martins et al., 2016; Marasco et al., 2021). Concerning biotic factors, the key role that plant genotype plays in shaping the microbial communities of leaf and fruit (Müller et al., 2015; Malacrinò et al., 2022) and root (Chialva et al., 2021) has been highlighted (**Table 3**). The effects of the plant phenological stage on the leaf-associated microbial community was analyzed as well (Abdelfattah et al., 2015; Kakagianni et al., 2023) (**Table 3**). Little is known about shifts in the olive microbiome caused by the presence of pathogens. Vergine and co-authors described significant changes taking place in the endophytic fungal and bacterial communities associated with olive leaves and branches during OQDS (Vergine et al., 2020). Moreover, evidence about the effect of *X. fastidosa* on the microbiome present in the olive xylem has been compiled during the last years (Morelli et al., 2017; Giampetruzzi et al., 2020; Anguita-Maeso et al., 2021b) (**Table 3**). Concerning the olive root microbial community, perturbations due to the infection by *V. dahliae* have been examined using a metatranscriptomic approach of pre-existing data (Martí et al., 2020) and by high-throughput sequencing of the rhizosphere and root endosphere microbiota of two cultivars differing in VWO tolerance (Fernández-González et al., 2020a) (**Table 3**). Regarding the impact caused by the introduction of BCAs/PGPMs on the olive microbial community, our most recent results have been already mentioned above (Cardoni et al., 2023a).

### 5.3 The olive microbiome as reservoir of beneficial microorganisms helping the holobiont to confront biotic stresses

When testing plant-associated microorganisms up to 35% of them can show the capacity to *in vitro* inhibit the growth of pathogens (Berg et al., 2006). However, the efficacy displayed by promising antagonistic microorganisms under these conditions do not always translate to actual biocontrol under natural/field scenarios (Fürnkranz et al., 2009) (see also section 5.2). Pathogen suppression mediated by microbial BCAs can be based on several mechanisms (e.g. antibiosis, competition, parasitism, etc.) (see, for instace, Nishad et al., 2020; Berg et al., 2021). Furthermore, some PGPM have been identified to be able to modify the quantity and composition of root exudates (Matilla et al., 2010; Florio et al., 2019) or to “recruit” new members of the soil microbiota to provide beneficial services to the plant (Santoyo, 2022). For example, the recruitment from the rhizosphere of bacteria and fungi with antagonistic activity toward *V. dahliae* was suggested to be favored in olive by the presence of *P. simiae* PICF7 or *P. polymyxa* PIC73 (Cardoni et al., 2023a). The success of any given BCA primarily depends on its competence to colonize and persist in the target niche. Several microbial traits (i.e. motility, biofilm formation, chemotaxis to root exudates and mucilage) offer a selective advantage for root colonization (Gómez-Lama Cabanás and Mercado-Blanco, 2020). For example, the loss of the ability to colonize the interior of olive roots by mutants of the BCA *P. simiae* PICF7 impaired in biofilm formation has been demostrated (Montes-Osuna et al., 2021a).

Regarding diseases affecting olive aerial organs some studies on the use of beneficial components originating from the indigenous microbiota are available. For instance, bacteria isolated from symptomatic olive plants were identified as promising candidates for biocontrol against *P. savastanoi* pv. *savastanoi* (Krid et al., 2012; Maldonado-González et al., 2013; Mina et al., 2017; Filiz Doksöz and Bozkurt, 2022), and diseases caused by pathogenic fungi. In the latter cases, inhibitory effects on conidial germination (Salman, 2017) and growth (Al-khatib et al., 2010) of *V. oleaginea* have been reported. Similarly, other BCAs can constrain growth, sporulation and germination of *C. acutatum*, and produce abnormal development of the pathogen’s hyphae (Preto et al., 2017) (**Table 4**).

**Table 4:** Examples of biological control agents originating from the olive-associated microbiota.

BCA/consortium components name	Target pathogen	Proposed mechanism of action	Organ of origin	Cultivar of origin	References
Olive aboveground part					
*Bacillus subtilis* F-1, F-4	*Pseudomonas savastanoi*	Antibiosis and induction of systemic resistance	Leaf	Chemlali	Krid et al., 2012
*Frondihabitans sp* *Paenibacillus sp*	*Pseudomonas savastanoi*	Production of indoleacetic acid and siderophore	Leaf		Mina et al., 2017
*Bacillus megaterium* HZEP7 *Bacillus subtilis* HZEN1 *Pseudomonas koreensis* HZEN27	*Pseudomonas savastanoi*	Production of siderophore and protease	Shoot and root		Filiz Doksöz and Bozkurt, 2022)
*Pseudomonas* ORS3 *Bacillus* BAT	*Venturia oleaginea*		Leaf		Salman, 2017
*Bacillus megaterium* NB-3 *Bacillus cereus* NB-4, NB-5, NBII *Bacillus subtilis* NB-6, HNEB-1 *Corynebacterium xerosis* NB-2 *Burkholderia mallei* NB-8	*Venturia oleaginea*	Production of siderophore	Leaf		Al-khatib et al., 2010
*Quambalaria cyanescens* *Epicoccum nigrum* *Aspergillus brasiliensis* *Chondrostereum purpureum* *Chaetomium globosum* *Aspergillus westerdijkiae* *Aspergillus* sp. 1	*Colletotrichum acutatum*	Production of wall cell degradation enzymes	Fruit	Verdeal Transmontana and Madural	Preto et al., 2017
Olive belowground part					
*Bacillus licheniformis* *Enterobacter colcae*	*Fusarium solani*		Rhizospheric soil		Bouzoumita et al., 2020
*Trichoderma* *harzianum* Ths97	*Fusarium solani*	Induction of plant resistance, above all mechanisms related with ROS defence	Root		Ben Amira et al., 2017
25 species of *Glomus*, *Acaulospora, Gigaspora*, *Entrophospora* and *Scutellospora* (Kachkouch et al., 2014)	*Verticillium dahliae*	Induction of systemic resistance	Root	Picholine Marocaine, Picholine du Languedoc, Dahbia, Mesllala, Picual and Manzanille	Boutaj et al., 2019
*Claroideoglomus etunicatum*, *Rhizophagus prolifer, R. clarus, R. diaphanum, R. intraradices, Funneliformis mosseae, F. geosporum, Septoglomus constrictum, Diversispora versiformis, Glomus* sp1, *Glomus* sp2*, Glomus* sp3, *Glomus sp4, Glomus sp5, Acaulospora* *denticulata, A. spinosa, A. kentinensis, Acaulospora* sp1*, Acaulospora* sp2, *Acaulospora* sp3, *Acaulospora* sp4, *Entrophospora* sp1, *Gigaspora* sp1*, Gigaspora* sp2, *Gigaspora* sp3 and *Scutellospora* sp1	*Verticillium dahliae*	Induction of systemic resistance	Root		Boutaj et al., 2020
*Trichoderma asperellum* *Trichoderma hamatum* *Trichoderma harzianum*	*Verticillium dahliae*	Production of volatile compounds	Rhizospheric soil		Aleandri et al., 2015
*Bacillus amyloliquefaciens*	*Verticillium dahliae*		Leaf		Müller et al., 2015
*Bacillus velezensis* OEE1	*Verticillium dahliae*	Antibiosis	Root	Chemlali	Azabou et al., 2020
*Phoma* sp., *Pseudomonas simiae*, *Pseudomonas putida, Pythium oligandrum*, *Trichoderma* sp.*, Trichoderma atroviride, Fusarium oxysporum, Gliocladium roseum, Glomus intraradices, Paenibacillus* sp.	*Verticillium dahliae*	Induction of plant resistance and direct antagonism	Root, leaf, olive fermentation brine	Picual, Arbequina, Wardan	Varo et al., 2016
*Pseudomonas* spp. PICF1, PICF3, PICF4, PICF6, PICF7, PICF8 *Pseudomonas putida* PICP2, PICP5	*Verticillium dahliae*	Production of siderophore, cyanide and pseudobactins	Root	Picual	Mercado-Blanco et al., 2004
*Pseudomonas simiae* PICF7	*Pseudomonas savastanoi* *Verticillium dahlilae*	Antibiosis, induction of plant resistance	Root	Picual	Maldonado-González et al., 2013 Gómez-Lama Cabanás et al., 2014; Gómez-Lama Cabanás et al., 2017; Martínez-García et al., 2015
*Pseudomonas* spp. PICF6, PICF7	*Verticillium dahliae* *Verticillium longisporum*	Production of volatile organic compounds	Root	Picual	Montes-Osuna et al., 2021a
*Pseudomonas* spp. PIC141, PIC25, PIC105	*Verticillium dahliae*	Production of siderophore and cyanide	Root	Picual	Gómez-Lama Cabanás et al., 2018a
*Paenibacillus polymyxa* PIC73, PIC167 *Bacillus* sp. PIC28	*Verticillium dahliae, Rosellinia, Phytophthora cinnamomi, Pseudomonas savastanoi, Colletotrichum nymphaeae* and *C.* *godetiae*	Production of protease, cellulose, siderophore, 2,3-butadenediol, chitinase, amylase and xylanase	Root	Picual	Gómez-Lama Cabanás et al., 2018b

Concerning the belowground level, bacteria (Bouzoumita et al., 2020), fungi (Ben Amira et al., 2017) and AMF (Castillo et al., 2006; Porras-Soriano et al., 2006; Boutaj et al., 2019; Boutaj et al., 2020) isolated from olive roots, showed *in vitro* antifungal activity against *F. solani* and *V. dahliae* (**Table 4**). Other studies, although not reporting a direct control of the pathogen by AMF, described increases in the number of shoots, leaves and plant height in plants inoculated with these fungi (Porras-Soriano et al., 2006; Chatzistathis et al., 2013; Khrieba et al., 2019). Against *V. dahliae* the effectiveness of the filamentous fungal genus *Trichoderma* (Aleandri et al., 2015) and *Bacillus* members (Müller et al., 2015; Azabou et al., 2020), both isolated from olive plant, were also reported (**Table 4**). *In vitro* and *in planta* antagonistic ability against *V. dahliae* has been thoroughly investigated for *P. simiae* PICF7, an indigenous inhabitant of olive roots (Mercado-Blanco et al., 2004; Martínez-García et al., 2015; Montes-Osuna et al., 2021a). However, no protective effect was found for this strain to mitigate drought and salt stress (Montes-Osuna et al., 2021b). Previously, this and other 28 strains of bacteria and fungi isolated from olive organs were tested for their *in vitro* antagonistic activity against *V. dahliae* (Varo et al., 2016) (**Table 4**). Another promising beneficial rhizobacteria originating from the olive associated microbiota that showed antagonist *in vitro* activity against a broad range of olive pathogens, including the causal agent of VWO, is *P. polymyxa* PIC73 (Gómez-Lama Cabanás et al., 2018b; Cardoni et al., 2023a). Many other examples could be brought to this section and some of them are summarized, not exhaustively though, in **Table 4**. This highlights that the microbial community associated with the olive tree is a powerful source of microorganisms yet to be identified, characterized and qualified as stress protectants. Besides, while some of them are (so far) difficult to isolate and cultivate, they could play the role of keystones within the olive microbiome, interacting with the host genetic machinery to prepare the holobiont to confront upcoming and/or persistant stresses (Fernández-González et al., 2021). Interestingly enough, (some) results mentioned above could fit the proposed ‘plant microbiome rewilding’ hypothesis (Raaijmakers and Kiers, 2022). That is to say, the reinstatement of key members of the original microbiota which could have been lost during domestication (e.g. breeding) and industrialization (e.g. use of pesticides and fertilizers) processes, can enhance the health of both plants and animals.

## 6 Concluding remarks

A growing number of studies have described the abiotic and biotic stresses affecting olive cultivation worldwide, including emerging constraints related with climate change scenarios and aerial and terrestrial pollution. In contrast, only a few of them have considered the effects on the olive-associated microbiome. Thus, the impacts over the microbial component of the olive holobiont have remained largely unknown, at least until very recently. The information available, however, is still fragmentary. Some studies are only focused on specific components of the microbiota (i.e. AMF, bacteria, archaea), and/or related only to a specific plant organ (e.g. fruit, leaf, branch). Besides, many of them have been carried out considering only culture-dependent methods that did not allow the description of the whole community (**Table 3**). The increasing number of studies describing the entire bacterial and fungal communities associated with different olive organs now raised important questions yet to be solved. For instance, to name a few of them, why the genotype-driven effect is stronger in olive fruits than in leaves or flowers? What are the consequences, as for stress resistance concerns, of the greater relative abundance of PGPR in olive cultivars classified as VWO-tolerant? Which is the role of *Actinophytocola* taxa in host defence mechanisms acting at the belowground level?

The interactions taking place among members of the olive microbiome are another aspect that deserves attention and that has been scarcely investigated. The few studies so far available have focused on the analysis of the alterations in the olive microbiota co-occurrence networks at very specific situations: the stress caused by the inoculation with pathogens and the impact of the introduction of BCAs. In contrast, the information about the consequences of abiotic stresses is null on this regard. These studies allow unveiling key roles that some specific keystone taxa seem to play in the olive holobiont’s response to biotic stress, and underline the need to investigate their functions at deeper levels. For example, the role of some keystone species in defense response against *V. dahliae* and *X. fastidiosa*, as well as the vertical transmission through generations of specific keystone bacterial genera related with olive growth promotion, are questions already suggested that need to be further addressed in future studies. The impact of selected BCAs on the olive root-associated microbiota networks has been described when applied individually. However, new strategies in biocontrol aim to the use of consortia of microorganisms or synthetic communities (SynComs) whose components work coordinately to achieve a more effective control of a given plant pathogen than that achieved by a single BCA. It could be expected that the impact caused by the introduction of a group of microorganisms would not be the same than that occurring when a single beneficial bacterium or fungus is inoculated. Therefore, the study of the impacts caused by the inoculation of microbial consortia, SynComs, or even entire microbiomes, over the natural, preexisting microbiota of a target holobiont is a research field yet to be explored. The powerful omics and bioinformatics tools available will yield exciting outcomes in this regard. Even though the microbial communities associated with olive leaves and roots showed the ability to rearrange and return to the original state after confronting a biotic stress (Fernández-González et al., 2020a; Vergine et al., 2020; Cardoni et al., 2023a), the potential ecological impacts on the entire holobiont remain unknown. A comprehensive knowledge of the interactions among the constituents of the olive microbiome and of the effects caused on them by different stresses, could help us to increase the resistance/resilience of the indigenous communities against external perturbations. Moreover, this information will be extremely helfpful to develop new inoculants with superior survival ability and low or null environmental impact, and to identify new PGPM with enhanced capabilities.

It must be emphasized that the relation between the host and its microbiome is mutual and dynamic. While this has been studied in some plant species (Kudoyarova et al., 2017; Bergelson et al., 2018; Grover et al., 2021; Herms et al., 2022), the complex and multiple dialogues established in the olive holobiont has not started to be unveiled until very recently, and only at the root level (Fernández-González et al., 2021). However, other aspects remain unknown. For example, no studies have been carried out on the recruitment of rhizobacteria by the olive plant through root exudates, or no GWAS have been performed to identify candidate genes associated with microbiome diversity and structure. Likewise, it is still unknow the role that specific compounds (e.g. VOCs, phytohormones) produced by PGPMs associated to a given olive organ have, at least to some extent, on its morphology, development and functionality.

**Figure 4:**
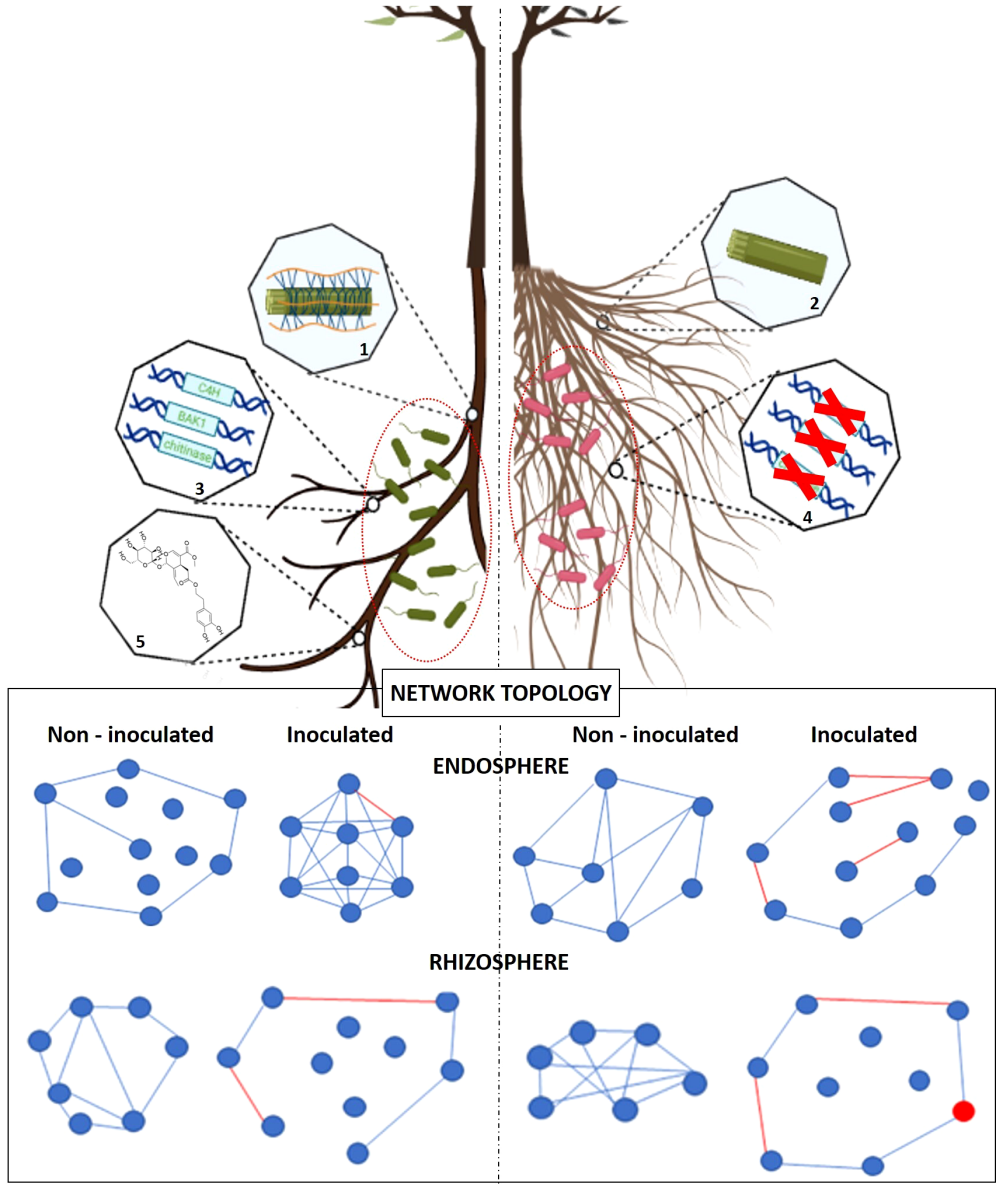
Schematic representation of the analysis of the interaction between the root system of VWO-tolerant (left) and VWO-susceptible (right) olive cultivars and *Verticillium dahliae* under the holobiont perspective. The differences in root system architecture are symbolised by diverse representation of the root morphology. The high (VWO-tolerant plants) (1) or low (VWO-susceptible plants) (2) content of lignin is shown by blue lines (or their absence) over the lignocellulose structure (drawn as a green cylinder). The faster (VWO-tolerant plants) (3) and slower/absent (VWO-susceptible plants) (4) expression of defence genes, in presence of the pathogen, is represented by DNA helices (marked with a red X in the case of slow/null expression). The high content in secoiridoids in VWO-tolerant plants (5) is represented by the chemical formula of oleuropein. The different taxonomical composition of the VWO-tolerant and VWO-susceptible associated microbial communities is shown in green and red, respectively. In the bottom part, a schematic, simplified representation of the main effects of *V. dahliae* inoculation in root endosphere and rhizosphere microbial networks is shown. Red edges represent negative interactions between modules (solid circles) and the red circle represents the module that includes the pathogen. Based on Cardoni et al. (2022) and Fernández-González et al. (2020a), and partially generated with BioRender.com.

In this review we aimed to underline the importance to consider the holobiont context when confronting stresses than can affect the olive tree. A useful model of this holistic approach can be exemplified by our findings on the interaction between the olive root system and *V. dahliae*, and the multilevel (i.e. morphological, biochemical, genetic and microbiological) responses taking place in this part of the holobiont (**Figure 4**). First of all, the architecture of the root system is the first feature determining the predisposition of a given olive cultivar to be infected and colonized by *V. dahliae*. Indeed, less surface and length and low number of tips and forks usually present in the roots of the VWO-tolerant cultivars make them less exposed to be contacted by pathogen propagules (Cardoni et al., 2021; 2022). Secondly, larger diameter of thin roots and higher basal content of lignin and secoridoids, make these cultivars more difficult to by colonized by *V. dahliae*. Therefore, the biochemical composition of the roots is another important characteristic contributing to explain olive tolerance to this pathogen (Cardoni et al., 2023b). The third important feature of the VWO-tolerant cultivars is their speed in sensing the presence of the pathogen and activating their genetic defense-response machinery (Cardoni et al., 2022). Another main trait influencing VWO tolerance is related with the taxonomical composition of the olive root-associated microbiota and the interactions taking place among its members (Fernández-González et al., 2020a). Indeed, few differences in the relative abundance of specific major taxa, mainly in the root endosphere compartment, seem to contribute in determining the success or failure of olive varieties to cope with the pathogen. Nevertheless, more decisive is the topology of the root microbiome networks, especially their changes when the pathogen is present. In conclusion, the importance to adopt a multidisciplinary approach to in depth comprehend, and to successfully manage, any biotic or abiotic stress affecting the olive holobiont is instrumental and must be highlighted. Our recent outcomes were focused on the olive root compartment, but the same approach should be extended to the whole plant considering the exchange of compounds, genetic material and microorganisms that constantly take place among the different compartments of the holobiont. Only keeping in mind this perspective would it be possible to develop truly efficient and sustainable strategies for the control of both traditional and emerging stresses. Moreover, novel information and tools could be incorporated in breeding programs for the development of olive varieties tolerant/resistant to abiotic stresses (i.e. drought, salinity, temperature, etc.) so far less investigated from this holistic perspective.

## Author contributions

MC: Conceptualization, Writing – original draft. JM-B: Conceptualization, Funding acquisition, Supervision, Writing – review & editing.
